# A Review of Characterization and Modelling Approaches for Sheet Metal Forming of Lightweight Metallic Materials

**DOI:** 10.3390/ma16020836

**Published:** 2023-01-15

**Authors:** Yong Hou, Dongjoon Myung, Jong Kyu Park, Junying Min, Hyung-Rim Lee, Ali Abd El-Aty, Myoung-Gyu Lee

**Affiliations:** 1 Department of Materials Science and Engineering & RIAM, Seoul National University, Seoul 08826, Republic of Korea; 2 Hwashin Co. Ltd., Yeongcheon 770-280, Republic of Korea; 3 School of Mechanical Engineering, Tongji University, Shanghai 201804, China; 4 Mechanical Engineering Department, College of Engineering at Al Kharj, Prince Sattam Bin Abdulaziz University, Al Kharj 16273, Saudi Arabia

**Keywords:** lightweight metallic materials, sheet metal forming, constitutive modelling, numerical simulation, formability

## Abstract

Lightweight sheet metals are attractive for aerospace and automotive applications due to their exceptional properties, such as low density and high strength. Sheet metal forming (SMF) is a key technology to manufacturing lightweight thin-walled complex-shaped components. With the development of SMF, numerical simulation and theoretical modelling are promoted to enhance the performance of new SMF technologies. Thus, it is extraordinarily valuable to present a comprehensive review of historical development in SMF followed by state-of-the-art advanced characterization and modelling approaches for lightweight metallic materials. First, the importance of lightweight materials and their relationship with SMF followed by the historical development of SMF are reviewed. Then, the progress of advanced finite element technologies for simulating metal forming with lightweight alloys is covered. The constitutive modelling of lightweight alloys with an explanation of state-of-the-art advanced characterization to identify the constitutive parameters are presented. Then, the formability of sheet metals with major influencing factors, the techniques for measuring surface strains in SMF and the experimental and modelling approaches for determining the formability limits are clarified. Finally, the review is concluded by affording discussion of the present and future trends which may be used in SMF for lightweight metallic materials.

## 1. Introduction

**Figure 1:**
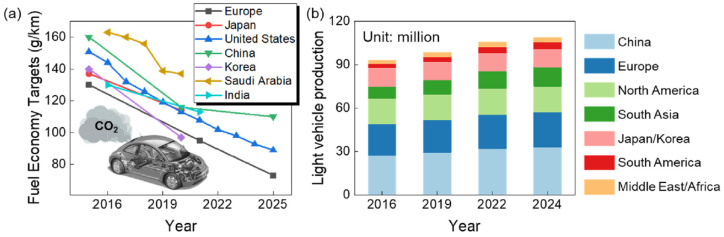
The current and future impact of lightweight materials in the automotive industry on (**a**) fuel economy and (**b**) production for the major markets. Reprinted from Ref. [9], open access.

**Figure 2:**
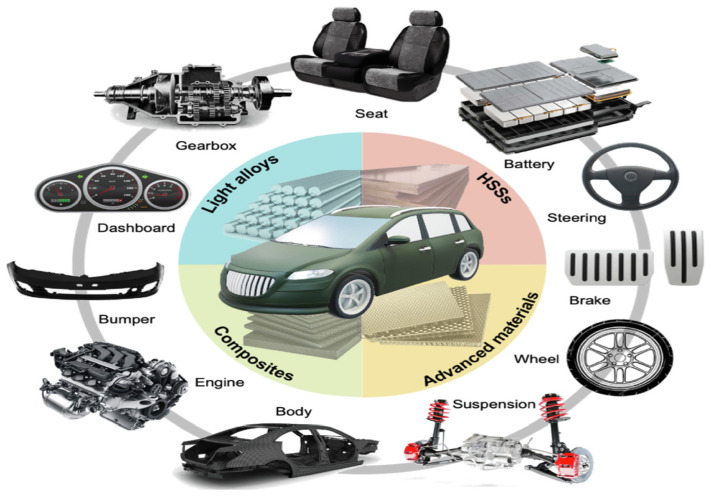
A schematic representation of the applications of lightweight materials in the automotive industry. Reprinted from Ref. [9], open access.

**Figure 3:**
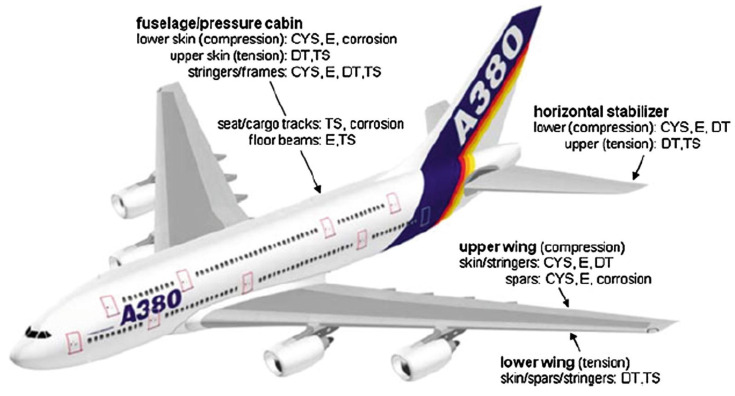
The application of Al alloys in aircraft. Reprinted from Ref. [10] with permission. Copyright 2018, Elsevier.

Lightweight metallic materials such as aluminium (Al), magnesium (Mg) and titanium (Ti) alloys have gained significant attention and are at the cutting edge of research and development activities in the automotive and aerospace industries for enhancing fuel economy and thus reducing gas emissions (Figure 1) [1,2,3,4,5,6,7,8,9,10]. Therefore, it is crucial to develop new lightweight materials and propose new manufacturing processes to produce high-quality lightweight components and simultaneously fulfil safety requirements. Figure 2 and Table 1 summarize the applications of lightweight materials in the automotive industry and Figure 3 presents the applications of Al alloys in the aircraft industry.

**Figure 4:**
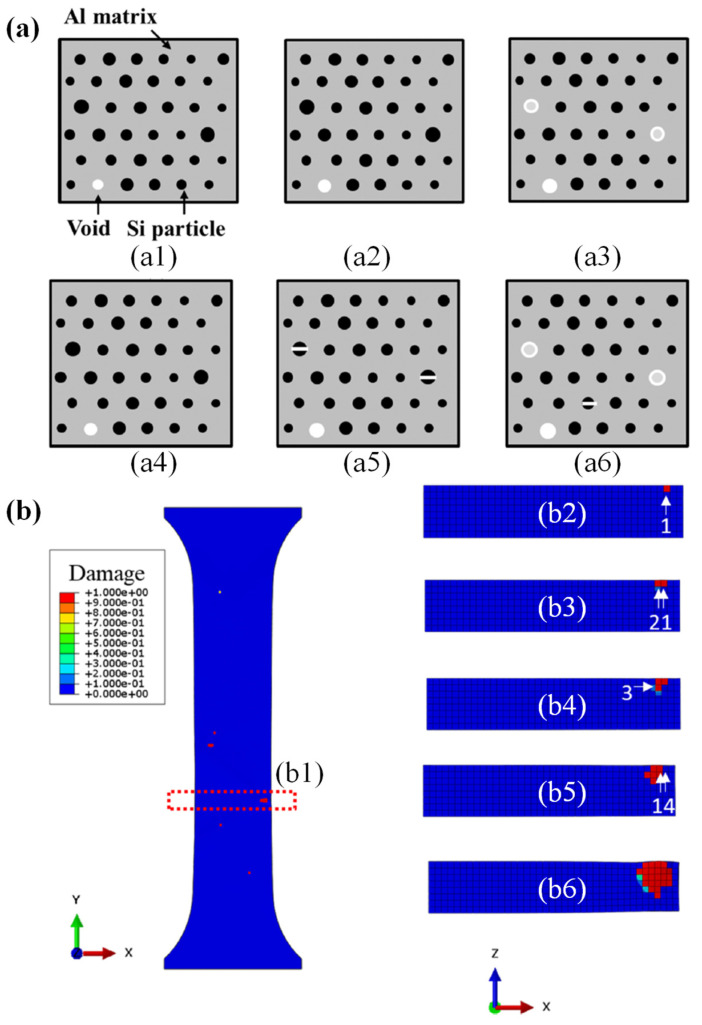
A schematic description of coupling numerical simulation with fracture model to predict internal cracks. (**a**) The damage evolution at the material point: (a1), (a2) and (a3) are the start of time step increment; (a4)–(a6) are the post-time steps of (a1), (a2), and (a3), respectively. (**b**) The damage value contour of simple tension simulation: (b1) shows the whole model in the X–Y plane, and (b2)–(b6) shows damage propagation in the Z–X plane through time at the red marked region in (b1). Reproduced with permission [24]. Copyright 2022, Elsevier.

**Figure 5:**
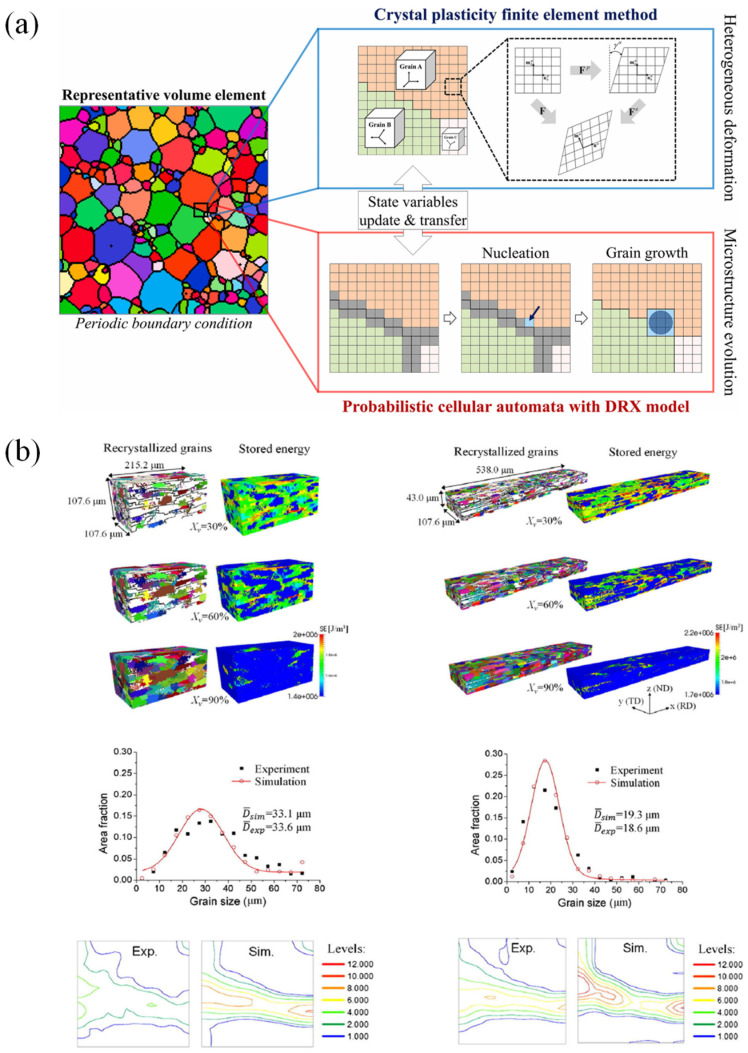
(**a**) A framework for the integrated CPFEM approach [31] and (**b**) an example of numerical simulations coupled with the microstructure evolution [30]. Reproduced with permission [31]. Copyright 2022, Elsevier; Reproduced with permission [30]. Copyright 2022, John Wiley and Sons.

Currently, due to the development of computing technologies, numerical simulation has gained much attention and is used widely for simulating SMF processes [11]. Many industrial sectors and research development centers have employed numerical simulations to optimize complex processes [12,13,14]. Numerical simulation can predict the forming forces, deformation behavior of sheet metals, sheet thickness, temperatures, stresses distributions [15,16,17,18], springback [19,20,21,22] and potential cracking and wrinkling [19,23,24], as introduced in Figure 4. Furthermore, numerical simulation has also been applied for characterizing the behaviors of materials at the microscale, which is based on crystal plasticity (CP) modelling [25,26,27]. CP has been used to predict microstructure evolution during material deformation [28,29,30,31] as depicted in Figure 5. Besides, the conventional numerical approach based on plasticity theory was coupled with damage models for predicting the flow behavior of metallic materials [32] and their forming limits [33,34] under complex forming conditions.

In manufacturing automobile body components, known as body-in-white (BIW) manufacturing, SMF has been considered among the most crucial manufacturing technologies [35]. In recent years, great efforts have been made by universities and manufacturing companies to propose new SMF technologies to meet the customers’ and manufacturers’ requirements. In SMF processes, thin metallic sheets are stretched into the desired product shape using different tools without excessive thinning or wrinkling [36,37,38]. Several key factors should be considered during developing new SMF technologies, which affect the final shape and the quality of the products. Insufficient consideration of these factors will cause excessive thinning, wrinkling, buckling and tearing in the components. These factors include forming speed, temperature, friction conditions, mechanical properties of materials and the geometrical details of forming tools [39,40,41,42,43,44,45].

To date, various numerical simulation approaches have been developed for SMF processes. They can be classified as the discrete element method (DEM) [46,47], boundary element method (BEM) [48], finite volume method (FVM), finite difference method (FDM) [49], finite element method (FEM) including crystal plasticity finite element (CPFEM) [50,51,52,53], extended finite element (XFEM) [54], multi-grid and mesh-free methods [55], fast Fourier transformation (FFT) [56] and arbitrary Lagrangian-Eulerian (ALE) [57,58].

Previously, many researchers have discussed the progress of SMF simulation from different aspects and there is a remarkable literature containing discussions of the importance of SMF simulations. For instance, Kaftanoglu and Tekkaya [59] briefly described a complete numerical solution for the axisymmetric deep drawing problems. Then, Makinouchi [60] introduced applications of FE simulations of SMF in several industrial sectors. He offered an outstanding literature review of existing SMF simulation codes and several examples for different industrial sectors. Makinouchi et al. [61] presented the current status and progress of SMF simulations in industries in Japan, Europe and the United States. Tekkaya [62], Tisza [63] and Wenner [64] presented developments and progress in SMF simulations in terms of methodologies, type of element and the available FE software before 2005. Ahmed et al. [65] presented the progress of SMF simulations from several points of view, such as continuum and shell approaches, material and geometrical non-linearity and frictional contact. They also discussed error estimations, error projections and adaptive mesh-refinements in SMF simulations. Banabic [66] discussed the principles of plasticity theory in SMF processes. He detailed the formability of sheet metals and presented various mathematical modelling techniques for forming limit predictions. Another important role of simulation is to quantitatively evaluate the influence of forming process parameters on the mechanical properties of formed parts through the calibrated simulation model to provide effective guidance for subsequent forming process optimization [67,68,69]. There have been numerous studies and reviews of SMF simulations, from fundamental plasticity theory to the optimization of FE models [31,70,71,72].

**Figure 6:**
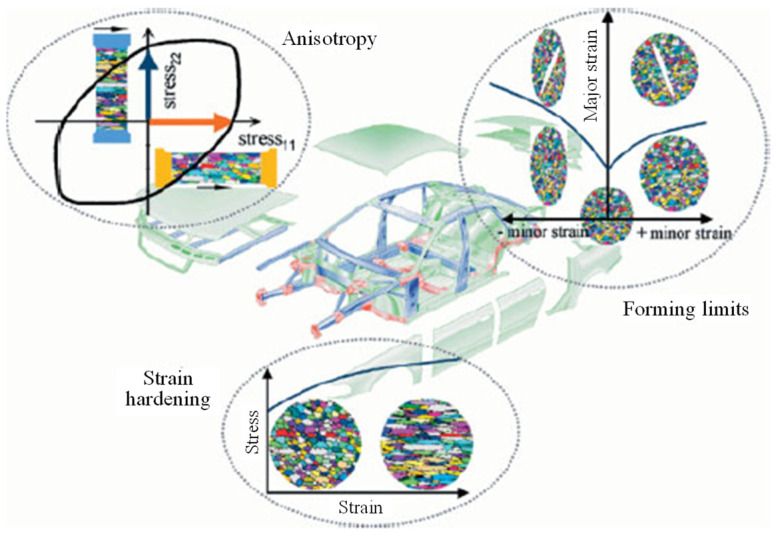
A schematic diagram describing the materials input date used for modern FE technologies to optimize SMF processes. Reprinted from Ref. [73] with permission. Copyright 2002, John Wiley and Sons.

As described above, there has been significant progress on SMF technologies including FE simulations, but only limited literature can be found regarding a more comprehensive review of SMF technologies for lightweight metallic materials and their advanced characterization and modelling approaches. With this background, we tried to provide a review of the historical development followed by the state-of-the-art of advanced characterization and modelling approaches in the field of SMF with lightweight alloys. This review reports on the current status and future trends in experimental techniques to identify constitutive models for lightweight alloys. Theoretical and numerical models for evaluating formability are also presented along with the FE modelling approach to prove the anisotropic behavior of sheet metals [73] (Figure 6).

In addition to forming single-layer metallic sheets, investigations on bilayer or multilayer sheets have also attracted much attention for several special components, such as pouch batteries. Yanagimoto et al. [74] observed the enhancement of the bending formability of type-420J2 stainless steel sheets when they are composed into a multilayer metallic sheet with type-304. Rydz et al. [75] analyzed the shaping of bimetallic Al–Cu sheets in cup drawing tests. Kim et al. [76] predicted the forming limit curve of a three-layer AA5182-O/polypropylene/AA5182-O (AA/PP/AA) sandwich sheet based on the Marciniak-Kuczynski (M-K) model and strain-rate potentials. Microstructure, texture, anisotropy, formability and mechanical properties of a layered composite (Brass/IFS/Brass) at various annealing temperatures were investigated by Bagheri et al. [77].

**Figure 7:**
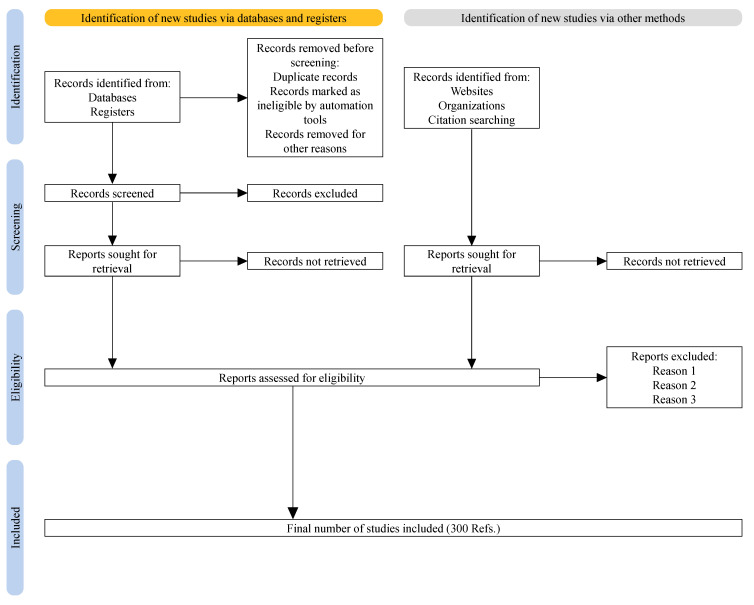
A schematic flowchart of PRISMA guidelines [78] used to prepare the current review.

**Figure 8:**
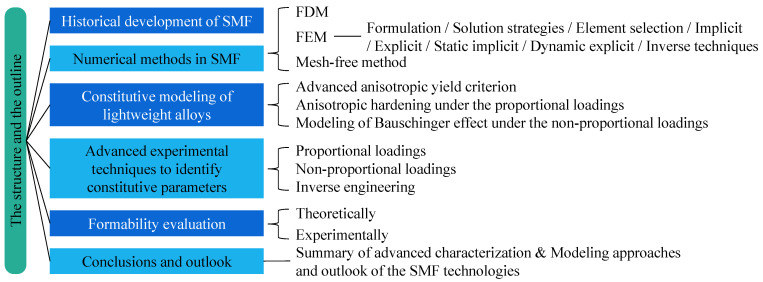
The structure of the current review.

The content of this review paper follows the PRISMA guidelines [78], as depicted in Figure 7. As summarized in Figure 8, we begin with a brief discussion of simulation methods in SMF. Then, progress in constitutive modelling and the experimental techniques used to identify the constitutive parameters are discussed in Section 3 and Section 4, respectively. Subsequently, the theoretical and numerical models used for formability evaluation are summarized in Section 5. Finally, the paper is concluded by providing discussion on advanced characterization and modelling approaches for SMF and the outlook for SMF technologies.

## 2. History of Numerical Methods in Sheet Metal Forming Simulation

Early-stage simulation of SMF was restricted to two-dimensional symmetric simple problems. In the 1960s, the first simulation of the SMF process was performed by the finite difference method (FDM), simulating a two-dimensional cylindrical cup manufactured via a deep drawing process [79]. Afterwards, many trials were performed in the 1990s to use FDM for three-dimensional problems, but not very successfully because of the complexity in applying complicated boundary conditions. The FDM was utilized to simulate the thermal effect in SMF processes [80].

The finite element method (FEM) is the key method for simulating SMF processes. Wifi [81] presented a FEA of axisymmetric elastoplastic circular blank sheets for deep drawing and stretch forming processes. Then, a general formulation of FEA was proposed by Gotoh et al. [82] for deep drawing technology based on the rigid-plastic material model, where the analysis was performed by the quadratic and fourth-order yield functions. Afterwards, Wang et al. [83] proposed a general FE approach for the stamping process and the presumed small thickness of the sheet metal based on membrane theory. Besides, it is assumed that the sheet obeyed rate the independent elastic-plastic material model with the J2 flow rule. They demonstrated that both rigid-plastic and elastic-plastic material models generated similar strain distributions at the material’s point unloading.

Tang et al. [84] extended the implementation of FE simulation from two-dimensional to three-dimensional via modelling deformed automotive body panels. Afterwards, Toh et al. [85] also introduced a general approach to 3D sheet metal simulation. In these simulations, either a static implicit or explicit method was utilized [64,65] with elasto-plasticity as a material model [66]. Later, deformation mechanics were introduced to DYNA3D FE software by Benson et al. [86]. Then, Belytschko [87] applied the dynamic explicit technique to FE simulation software. Massoni et al. [88] discussed the principle of replacing the draw bead with an artificial force and Wang et al. [89] considered viscous effects in their study. From the late 1990s, predicting springback accurately gained much attention from many researchers, which in turn affected the developments in sheet metal simulations to design and develop accurate, robust and efficient algorithms and solution methods.

The literature discussed several SMF simulation techniques [80,81,82,83,84,85,86,89,90,91]; for instance, Makinouchi [60,61] categorized formulations into three types, i.e., static implicit, dynamic explicit and dynamic implicit formulations. Besides, the solution frameworks were classified into incremental, one-step and large-step methods. These methods were investigated in detail by comparing different numerical algorithms [92,93,94]. For example, Banabic [66] categorized the simulation methods based on constitutive equations and motion description to flow method, static implicit (solid) method, rigid-plastic method, static explicit method and dynamic explicit method. The implicit method used an iterative strategy to solve linear system problems, which offered correct and unconditionally stable solutions for the simulations. Therefore, a larger time step could be used in the simulation but, because of the iterative procedures in the solution process, this needs longer computation time and a huge memory. When a huge element’s numbers are included in the deformation, it is difficult to achieve convergence. Nevertheless, the explicit technique needs less memory and computation time. Besides, it can be efficiently parallelized and convergence is easily acquired. On the other hand, this technique is stable only under certain conditions.

When extending simulation from the 2D to 3D SMF process, the shortcomings of the implicit technique were reduced by using a static explicit technique where the forward Euler scheme is used to integrate a set of equations [60,61,62,95,96,97,98,99,100]. Finn et al. [101] and Micari et al. [102] introduced a novel approach by coupling the advantages of both implicit and explicit techniques. In this novel framework, the explicit technique and implicit technique are used for simulating the forming process and springback, respectively.

The one-step technique was modified by applying the single time-step and the original blank sheet metals were determined from the final shapes of the deformed sheets. This is based on assumptions such as ignoring friction and neglecting the history of contact and linear strain path. This technique needs only a small computational time [103,104,105]. Lan et al. [12] expanded this technique for non-linear problems and Kim et al. [13] proposed a multi-step inverse approach based on it. Then, Tang et al. [14] introduced a multi-step inverse approach to simulate the stamping processes. One-step technique was accomplished by analyzing the node’s position, the thickness of the initial blank and the strain distributions of the initial configurations with respect to the final configuration. The multi-step approach constantly expands the one-step technique among two consecutive steps. Azizi [106] studies the implementations of the one-step method in SMF in terms of convergence speed, type of solution and the solution time of the equations. Na and Chen [107] coupled a quasi-one-step approach with the conjugate gradient technique.

As the simulation methods progressively gained attention in industrial applications, commercial software was developed accordingly. ABAQUS (from Dassault Systèmes Simulia Corp.) and LS-DYNA (from Ansys, Inc) are the general FE software extensively used in SMF simulations. On the other hand, specialized software such as OPTRIS (from DYNAMIC SOFTWARE), PAMSTAMP (from ESI Group) and AutoForm (from AutoForm Engineering GmbH) is also broadly used [66]. In spite of the momentous development in computing power, simulation results still do not fulfil industrial requirements. Thus, further studies were performed to develop the static implicit approach in the long term because the requirement for high efficiency in industrial applications is directing further investigations to improve the static implicit method [92].

The key factors affecting the simulation results are the element type and formulation. Therefore, various element types were applied in the simulation for different SMF processes. Membrane, continuum (solid) and shell (thick/thin) elements were typically utilized in SMF simulations. Membrane elements are used when the sheet’s bending radius is more than 20 times the sheet’s thickness. Thus, the shell element is used in the simulation of the deep drawing process instead of the membrane element [62]. On the other hand, the shell element is a poor choice for the simulations when it is required to describe the deformation in the thickness direction [108]. Solid elements can describe through-thickness plastic deformation; therefore, they are an excellent choice to simulate blanking and hydroforming [97]. This leads to the development of 3D solid-shell and solid elements to simulate the SMF process if the deformation in the thickness direction is critical [108,109,110]. For deep drawing simulation, Menezes et al. [109] proposed a 3D iso-parametric element with selective reduced integration. For simulating springback, Papeleux and Ponthot [111] proposed an enhanced assumed strain (EAS) element and compared it with other elements. Furthermore, a 4-nodes tetrahedral element and an 8-node hexahedron element are also applied for simulating SMF [112,113,114]. Currently, Chung et al. [108] investigated the feasibility of using tetrahedron-MINI elements to simulate single and multi-layer SMF.

Along with conventional FEM, a meshless or meshfree approach was also applied in the simulation of SMF processes [115,116,117,118]. Some researchers introduced a meshless formulation for SMF simulations via an SCNI approach, which overcame the limitations of the Galerkin-based meshless approach [119]. Besides, others expanded the SCNI approach and applied it for simulating springback [119]. Liu et al. [120] used a meshfree approach in their study for simulating hemisphere drawing and deep drawing processes. Liu and Fu [121] proposed an adaptive multi-scale meshfree approach for simulating and analyzing springback at two scales. They determined low and high components of the effective strain via the integration of the decomposed low and high scales of the shape function of RKPM to a non-linear elastoplastic formulation. When identifying the high-strain areas, a suitable scheme of node refinement was applied to calculate the stress correctly, therefore predicting the springback. Then, they compared their experimental results with the results acquired from FEM, meshfree approach and adaptive meshfree approach and noticed that the adaptive meshfree approach’s results were very close to the experiment results. Liu et al. [120] applied a high gradient indicator to RKPM based on adaptive multiscale meshfree approach to simulate the deep drawing process with a high accuracy.

## 3. Constitutive Modelling of Lightweight Alloys

The constitutive model is the theoretical basis to describe the plastic deformation of lightweight sheet metals. The objective of this section is not to review all the existing constitutive models, but to provide an overview of recent advances regarding advanced yield criteria, flow rule and hardening law.

### 3.1. Advanced Anisotropic Yield Criterion

**Figure 9:**
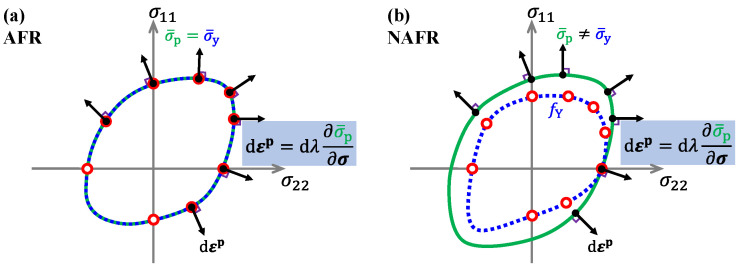
Constitutive model of plastic deformation under (**a**) AFR and (**b**) NAFR.

The phenomenological yield criterion provides an efficient way to describe yield behavior. Lightweight alloys show obvious plastic anisotropy, which was modelled by various anisotropic yield criteria [122,123,124]. The yield criteria can be divided into two categories (as shown in Figure 9). One is the yield criterion under the framework of the associated flow rule (AFR), where the yield function (${\bar{\sigma }}_{y}$) and the plastic potential function (${\bar{\sigma }}_{p}$) are identical, with the same parameters. The other type is the yield criterion under the non-associated flow rule (NAFR), where the yield function and the plastic potential function are two independent functions used to describe the yield stresses and the flow direction, respectively.

Most anisotropic plasticity models were developed based on AFR. Anisotropy parameters were introduced in the isotropic yield function to account for the plastic anisotropy. Hill [122] proposed a quadratic anisotropic yield function in 1948 based on the Mises yield criterion, which became one of the industry’s most widely used yield functions to describe anisotropic yielding behavior. Barlat and Lian [125] modified the isotropic Hosford 1972 yield function to account for in-plane anisotropy. Gotoh [126] proposed a fourth-order polynomial anisotropic yield criterion; other researchers also developed different polynomial equations to describe the yield surface of metallic materials [127,128,129]. However, identifying the material parameters of the polynomial yield function and the proof of the convexity of the yield surface are complicated. Therefore, a classical method using the linear transformation of the Cauchy stress tensor was developed to extend the isotropic yield function to the anisotropic yield function [123,130,131,132]. This method of linear transformation of the stress tensor can effectively improve the flexibility of the yield function and efficiently ensure convexity. The well-known 8-parameter Yld2k-2d model [123] is based on the AFR framework to capture the yield stress and strain rate ratio (r-values) by a linear combination of two functions based on a linear transformation. Cazacu and Barlat [133] proposed a representative theoretical framework for the second and third stress invariants. Based on the above-mentioned stress tensor linear transformation and representative theoretical framework, researchers have developed different advanced anisotropic yield criteria [134,135,136].

The anisotropic yield criteria mentioned above have been mostly used for sheet metals with body-centered cubic and face-centered cubic structures, such as high-strength steels or aluminium alloys. However, hexagonal close-packed (HCP) metals, such as magnesium alloys and titanium alloys, show obvious tension-compression asymmetry (TCA), i.e., strength differential (SD) effect under small plastic strain [137,138]. In addition, recent studies have found that third-generation advanced high-strength steels (AHSS) such as Q&P steels exhibit significant SD effect [21,139,140]. The above symmetric yield criterion cannot be used to describe the SD effect of special lightweight alloys. Several macroscopic yield criteria were developed to consider the SD effect, which is used in the SMF process to gain an accurate simulation for HCP metals. Cazacu and Barlat [141] proposed a new yield function to describe the SD effect of HCP metals. Cazacu et al. [142] developed an anisotropic asymmetric CPB2006 yield criterion using a linear transformation method for materials which is insensitive to hydrostatic stress. Khan et al. [143] proposed an asymmetric yield function related to the temperature and strain rate of Ti-6Al-4V alloy to describe the thermodynamic properties of Ti-6Al-4V in compression and tension loading. Yoon et al. [144] introduced the first stress tensor invariant to the asymmetric CB2004 yield function and proposed a stress-invariant-based yield criterion for pressure-sensitive metals. Hu et al. [145] linearly combined the cubic polynomial of the normalized third invariant and the stress triaxiality to improve the yield criterion in describing the SD effect with high flexibility.

Although the advanced yield functions under AFR can describe the yield behavior of metals and improve the accuracy of the finite element simulation, the forms of these functions become more complex, resulting in complicated parameter identification and high computing costs. To predict the yield stress and plastic flow of lightweight alloys with strong anisotropy, another approach is to use NAFR. Over the past decade, researchers generally believe that NAFR provides simple and efficient modelling to capture the anisotropic yielding and plastic flow of metallic materials. The model is formulated with a separate yield function and plastic potential function, both of which have a simpler function and convenient (analytical) parameter calibration. Stoughton [146] proposed a non-associative flow model based on the Hill48 function. With explicit parameter calibration, the Stouhton2002 model could accurately predict the uniaxial and biaxial yield stress with strong anisotropy. Later, plasticity models under NAFR were widely developed [147,148,149,150,151,152,153], as shown in Table 2, considering the non-quadratic feature and SD effect. The number of investigations and applications of NAFR is gradually increasing.

### 3.2. Anisotropic Hardening under Proportional Loadings

Due to the various hardening mechanisms, experiments show that hardening behaviors are stress-state-dependent and loading-orientation-dependent even under the proportional loadings [137,148,149,154]. These evolving yield surfaces cannot be captured by the anisotropic yield functions with isotropic hardening. Anisotropic hardening models can be divided into three groups. The first group captures the evolving yield surfaces at discrete levels of plastic deformation with an interpolation method. Hill and Hutchinson [157] studied the anisotropic hardening of the yield equation and the distortion of the yield surface. Aretz [158] established an anisotropic hardening model based on the variation of anisotropic coefficients with discrete equivalent plastic strain. The Yld2k-2d yield criterion was transformed to consider the anisotropic hardening by Wang et al. [159] and Cai et al. [160] using different evolving functions of equivalent plastic strain. The second approach to account for anisotropic hardening relies on introducing an evolving fourth-order tensor to modify the equivalent stress [161], or fourth-order tensors to the hardening function [162]. The third group of anisotropic hardening models was based on an analytical determination of anisotropic parameters without interpolation at discrete levels of plastic deformation. Stoughton and Yoon [148] proposed this kind of NAFR plasticity model based on the Hill48 quadratic function to capture anisotropic hardening, where four stress–strain curves in the different directions, 0°, 45°, 90° to RD and equi-biaxial tension were explicitly integrated into the yield criterion to describe the continuous change in anisotropy. Min et al. [149], Lee et al. [150] and Chen et al. [154] advanced this constitutive model scheme to obtain good agreement with measured data with a non-quadratic feature. Park et al. [151] and Hou et al. [155] further developed the CQN (coupled quadratic and non-quadratic) framework [150] to account for the SD effect. The pressure-sensitive function in Hou et al. [140,155,163] can directly employ the strain hardening curves along 0°, 45°, 90° to RD under uniaxial tension (UT) and compression (UC) and the equi-biaxial tension (EBT) condition. For the general yield criterion developed by Yoon et al. [144], the optimization method was used to determine the corresponding parameters. Hu et al. [156] proposed an analytical yield criterion based on Yoon’s yield function in 2014 [144] to describe anisotropy/asymmetry-induced distorted yield surface during deformation under proportional loadings. Most recently, Hou et al. [152] proposed a NAFR plasticity model with fourth-order polynomial functions (NAFR-Poly4) to accurately predict the anisotropic evolution of yield surfaces in sheet metals under plane strain loading. The current model accounts for the anisotropic yield stress under near-plane strain (NPS) states in the calibration step with an analytical parameter identification.

### 3.3. Modelling of the Bauschinger Effect under Non-Proportional Loadings

**Figure 10:**
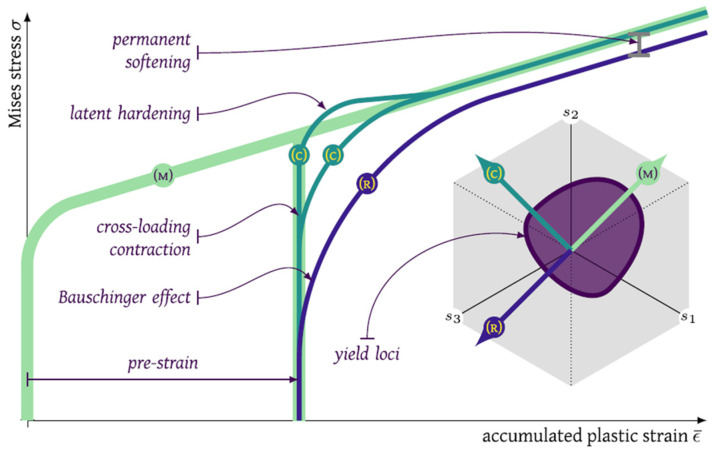
Graphical summary of the non-linear strain path effects: (M) monotonic represents no strain path change; (C) cross-loading is an orthogonal change of strain path in the deviatoric stress space; (R) reverse loading is an inversion of the reference direction. Reprinted from Ref. [166] with permission. Copyright 2022, Elsevier.

The Bauschinger effect refers to a material property of stress/strain characteristics under the non-proportional loadings, i.e., strain path changes (SPCs) due to microscopic activities. Studies show that sheet metals exhibit special hardening behavior, including the Bauschinger effect (early re-yielding), transient strain hardening and permanent softening under SPCs, as shown in Figure 10. This hardening behavior complicates the mechanical analysis of the forming process of lightweight alloys. To simplify the problem, the mechanical analysis of simple parts with traditional materials usually does not consider the Bauschinger effect-related hardening behaviors. However, the special hardening behaviour should be considered for the advance lightweight alloys under SPCs such as loading-unloading-reloading. Besides, numerous studies show that springback can be considerably affected by hardening behavior after reverse loading [164] or other complex strain paths [165]; thus, the Bauschinger effect-related hardening behaviors are required for springback simulations as one of the important factors.

Prager [167] proposed the earliest kinematic hardening model by introducing the concept of back stress to translate the yield surface to capture asymmetric plastic behavior. Armstrong and Frederick [168] proposed a kinematic hardening model for nonlinear back stress evolution and Chaboche [169] proposed a general nonlinear kinematic hardening model. Using this principle, researchers studied the kinematic hardening model of multi-yield surfaces. The most widely used two-yield surface model is the Yoshida-Uemori model [170], which can describe both hardening stagnation and elastic modulus degradation. Chaboche [171] reviewed nonlinear kinematic hardening models that have been used to accurately describe plastic mechanical behavior during reverse or cyclic loading. Many studies applied typical nonlinear kinematic hardening models to loading conditions involving arbitrary SPCs [172,173,174,175].

**Figure 11:**
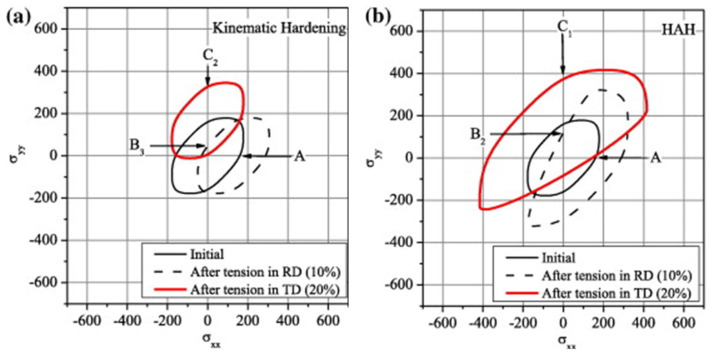
Yield loci during tension (up to 10% strain) followed by orthogonal tension (up to 20% strain) with (**a**) kinematic hardening and (**b**) HAH model. Reprinted from Ref. [182] with permission. Copyright 2012, Elsevier.

Another theoretical modelling approach to describe the Bauschinger effect related hardening behaviour during SPCs is the distortional hardening model proposed by Barlat et al. [176,177,178,179], namely the HAH model (Homogeneous yield function-based Anisotropic Hardening). This modelling method of distortion hardening rather than kinematic hardening (with back stress) is a viable option for describing the Bauschinger effect and other transient hardening phenomena. It introduced state variables such as microstructure deviators to record loading history [166]. Compared with the kinematic hardening model, the advantage of the HAH model is that the expression and parameter calibration of the isotropic and anisotropic hardening terms are independent of each other. The HAH model can be referred to as a framework because it can be used for any isotropic or anisotropic yield condition suitable for isotropic hardening and distorts the corresponding yield surface shape depending on the loading conditions. The HAH model can capture the measured Bauschinger effect with a fluctuating term in the cyclic loading path [180,181]. Figure 11 shows the schematic diagrams of the kinematic hardening model and the distortion hardening model: (1) In the kinematic hardening model, the center of the yield surface is translated in the stress space according to the loading path and the size of the yield surface is fixed or determined by the isotropic hardening equation; (2) in the distortion hardening model, the yield surface expands in the loading direction, while the contraction occurs in the opposite direction of the load path to capture the early yielding behavior and subsequent special hardening behavior.

HAH models have been continuously developed since initially proposed. They can better describe the evolution behavior of the yield surface under a wide range of SPCs [177,178,179,183,184]. The original HAH model (HAH2011) can only consider forward and reverse loading, such as tension-compression or shear-reverse shear [176]. Barlat et al. [177] extended the HAH model to account for the latent hardening during cross-loading, which accurately described the stress overshoot phenomenon of EDDQ steel in two-step UT tests. Lee et al. [185] combined the HAH2011 model and the QPE model: the HAH2011 model was utilized to capture complex plastic flow behavior, such as the Bauschinger effect, transient behavior, work hardening stagnation and permanent softening, while the QPE model reproduced unloading and nonlinear elastic behavior. Barlat et al. [178] enhanced the HAH model and this version improved the theoretical framework for the evolution of microstructure deviator and the distortion effects during cross-loading. He et al. [186] modified HAH2011 and introduced more parameters to describe the Bauschinger effect of the material in two-step UT tests. Lee et al. [187] proposed a modified distortional hardening model (HAH) that improved the description of differential permanent softening under various SPCs. Barlat et al. [179] developed the HAH model into the HAH2020 version, which incorporates the effect of hydrostatic pressure, manifesting as higher flow stress in UC than in UT. Compared to previous HAH models, the state variable evolution has been revised to improve the description of the material response when SPCs occur under pure cross-loading conditions. Reyne et al. [166] presented a new HAH-based framework, i.e., HEXAH, using an arbitrary number of microstructure deviators to describe a smooth evolution from one set of activated slip systems to another under abrupt SPCs.

In addition, some scholars proposed a hardening model that combines kinematic hardening and distortion hardening to describe the material response under SPCs. Francois [188] used distortion stress instead of Cauchy stress in the Mises yield criterion, which depends on two parameters, the back stress tensor and the scalar material constant. Feigenbaum and Dafalias [189], Rokhgireh et al. [190] and Qin et al. [191] proposed different approaches to achieve the combination of back stress (kinematic hardening) and distortion hardening. Holmedal [192] established a model that distorts the yield surface by flattening it in the opposite direction of loading. The model applies a pair of back stress tensors similar to the kinematic hardening model. Table 3 summarizes the constitutive models proposed to capture the Bauschinger effect under SPCs in terms of the adopted modelling strategies.

### 3.4. Application of Crystal Plasticity for Constitutive Modelling

**Figure 12:**
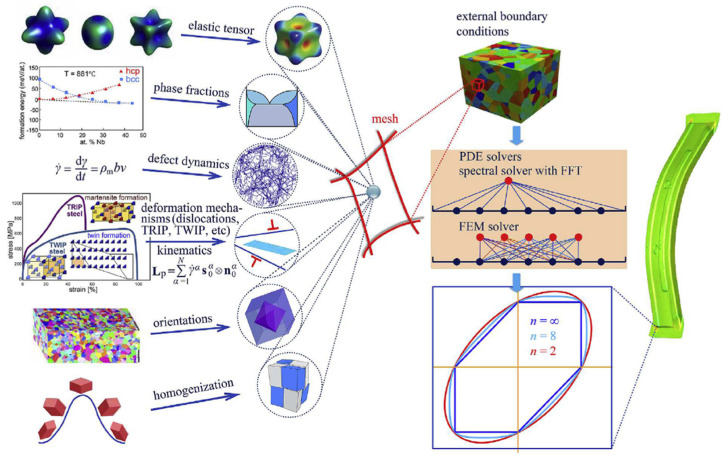
Yield surface calculated by DAMASK, a multi-scale and multi-physics CP modelling framework. Reprinted from Ref. [202] with permission. Copyright 2016, Elsevier.

**Figure 13:**
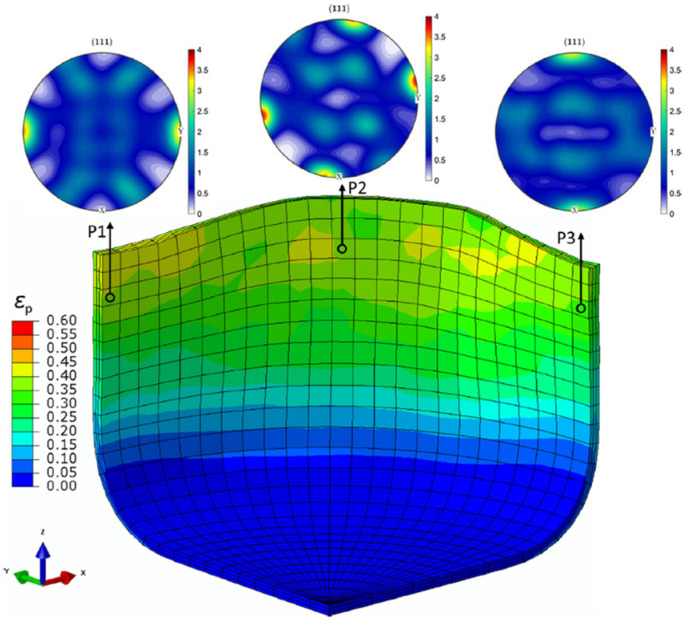
Equivalent plastic strain distribution and textures at three cup positions simulated with the evolving Yld2004-18p yield function calibrated from the CP model. Reprinted from Ref. [203] with permission. Copyright 2020, Elsevier.

As an advanced multi-scale modelling solution, CP is used to model the deformation behaviour of polycrystalline materials through the process of slip, twinning and phase transformation. The underlying crystal-level physical mechanisms, e.g., texture evolution and micromechanical field distribution, can be captured by CP models in addition to the macroscale stress–strain response. Wang and Wen [199] stated that CP models with high prediction capability are required for modern industry. The two widely used formulations of CP are: (1) that based on the FEM known as the crystal plasticity finite element method (CPFEM) [200] and (2) the spectral formulation, which is more computationally efficient based on the fast Fourier transform, but is for small strain formulation [201]. Figure 12 shows the various conceptual ingredients with different deformation mechanisms, phases, orientations and homogenization schemes that can be assigned to the same integration point in the DAMASK (Düsseldorf Advanced Material Simulation Kit) framework to provide the constitutive response at the mesoscale and predict plastic deformation at the component level [202]. Han et al. [203] developed an approach using CP-spectral based virtual experiments to update advanced anisotropic yield functions to realize a multi-scale model for formation of a of a 2090-T3 aluminium alloy sheet. Texture evolution at different positions of the cup was quite different, as shown in Figure 13.

Several advanced CP models were proposed to describe the constitutive behavior of magnesium alloy sheets (as HCP crystals) considering plastic anisotropy induced by the texture and the critical resolve shear stress (CRSS) ratio between available slip/twin systems [204,205,206,207]. Qiao et al. [208] proposed a simple empirical equation to model the twinning kinematics of magnesium alloy sheets. Recently, Shi et al. [137] reviewed the physics-based mesoscale modelling for the anisotropy of magnesium alloys. The mechanical responses of magnesium alloy sheets under different loading conditions, e.g., strain path changes [209,210,211,212], various strain rates [213,214,215] and elevated temperatures [216,217], were investigated by using CP models.

**Figure 14:**
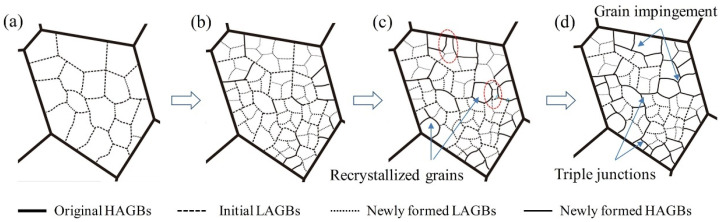
The proposed CDRX modelling approach by Chen et al. [220]: (**a**) initial microstructure before deformation; (**b**) microstructure change in the initial strain stage; (**c**) generation of recrystallized grains; (**d**) immobilization of recrystallized grain boundaries. Reprinted from Ref. [220] with permission. Copyright 2020, Elsevier.

CPFEM were employed to provide yield stresses and plastic strain rates under various loading conditions to identify the parameters of advanced yield functions, which were implemented in FE models to achieve accurate metal forming simulations of lightweight aluminium alloys [218,219]. In order to predict the effect of microstructure evolutions on the mechanical properties of aluminium alloys during the thermo-mechanical process, Chen et al. [220] proposed an integrated CP-continuous dynamic recrystallization (CDRX) framework, as illustrated in Figure 14. Recently, the modelling approaches of DRX for aluminium alloys during hot working were further developed [221,222].

**Figure 15:**
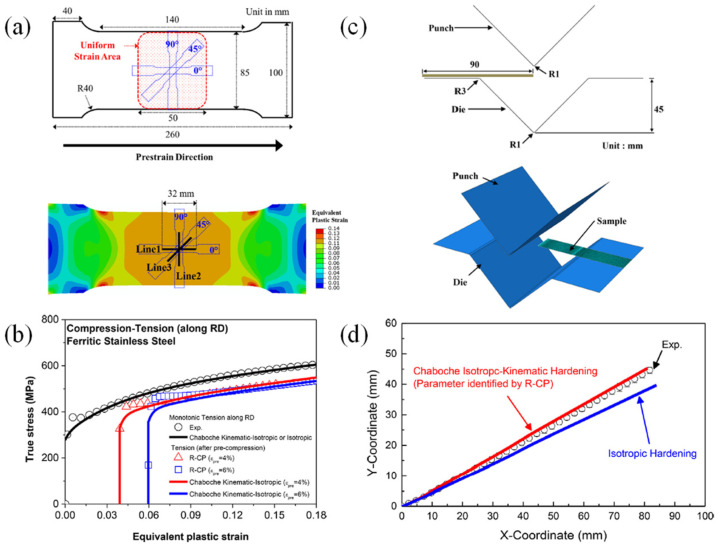
A CP approach based on a three-component dislocation density model to predict non-proportional anisotropic hardening behavior of ultra-thin sheet metals: (**a**) dimension of large specimen for the prior tension, ASTM E8 sub-size specimens for the second tension, and simulated equivalent plastic strain map after 25 mm tension; (**b**) CP predicted stress-strain curves of 0.1 mm thick ferritic stainless steel sheet during compression-tension along the rolling direction and comparison with Chaboche kinematic hardening model prediction; (**c**) dimension of V-bending test for 0.1 mm thick ferritic stainless steel sheet and FE model for V-bending test, and (**d**) comparison of springback profiles after V-bending of pre-deformed sample. Reprinted from Ref. [238] with permission. Copyright 2019, Elsevier.

CP models were utilized to investigate the microstructure evolution of advanced high-strength steels [223,224,225,226,227]. The strain-induced martensite phase transformation is the key feature of the third-generation advanced high-strength steels (3GAHSS). Accurate microstructural modelling of phase transformation (from retained austinite to martensite) by CP is critical to exploit new QP steels for automotive light-weighting [228,229,230,231,232]. Recently, a thermodynamically consistent constitutive model based on rate-dependent CP was developed to predict the stress, temperature and retained austenite evolution responses of a QP3Mn alloy over a wide range of strain rates and temperatures [232], where plastic slip and transformation kinetics laws were proposed to account for the temperature, strain and orientation-dependent mechanical behavior. Nowadays, the application of CP models in predicting the constitutive behaviors of lightweight alloys under complex loading conditions has drawn significant attention [233,234,235,236,237]. As shown in Figure 15, Bong et al. [238] proposed a CP approach based on a three-component dislocation density model as a virtual experimental model to accurately predict the non-proportional anisotropic hardening behavior of ultra-thin sheet metals. The validated model can predict the stress-strain curves under tension-compression loading, which are difficult to measure by mechanical experiments due to premature buckling for ultra-thin sheet metals.

## 4. Advanced Experimental Techniques to Identify the Constitutive Parameters

**Figure 16:**
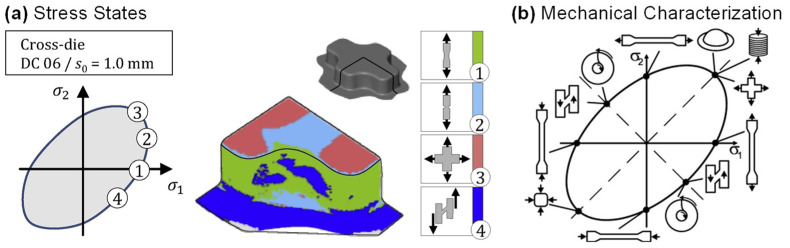
(**a**) Various stress states in deep drawing of a cross-cup. Reprinted from Ref. [240] with permission. Copyright 2018, Elsevier; (**b**) overview of testing methods for sheet metal characterization under different stress states. Reprinted from Ref. [241] with permission. Copyright 2014, Elsevier.

The advanced constitutive models require specific mechanical characterization methods to calibrate the parameters. A large amount of accurate and representative experimental data is an important input for the popular data-driven constitutive models [239]. The application of digital image correlation (DIC) technique in measuring strain field improved the accuracy of characterization experiments for sheet metals. It also promoted the development of various advanced characterization techniques [240]. The key to the experimental characterization of the yield surface evolution behavior is to use experimental equipment and systems to realize the plastic deformation of sheet materials under different typical stress states (as shown in Figure 16). Typical stress states include uniaxial tension (UT), uniaxial compression (UC), plane strain (PS), equal-biaxial tension (EBT) and simple shear (SH). For thin plate materials, it is generally considered to be in a state of plane stress during the forming process. For mechanical characterization, when the specimen is loaded at different angles from the rolling direction of the sheet metals, the material anisotropy can be investigated.

### 4.1. Proportional Loadings

#### 4.1.1. Uniaxial Tension (UT)

The UT test is the simplest method for sheet metals and an international testing standard has been established [242]. The UT test combined with DIC provides a quick tool to accurately obtain the basic mechanical properties of sheet metals, such as Young’s modulus, yield strength, tensile strength, uniform elongation, total elongation and r-values. These parameters work as significant engineering indicators for lightweight alloys. The stress–strain curves measured from the UT test are important data for investigating the hardening behavior of lightweight alloy sheets. Some classical constitutive models embedded in the commercial finite element software are calibrated directly using the UT experimental data to predict the forming process with acceptable accuracy. However, stress–strain curves measured from the UT test tend to over- or underestimate hardening curves under other stress states, which highly depends on the selected yield criterion. Furthermore, the obtained stress–strain curve from the UT test is limited due to the localized necking of the UT specimens. In order to obtain an accurate description of the post-necking hardening behavior, the method of curve fitting is adopted based on the chosen hardening law, e.g., Swift or Voce hardening laws.

#### 4.1.2. Uniaxial Compression (UC)

**Figure 17:**
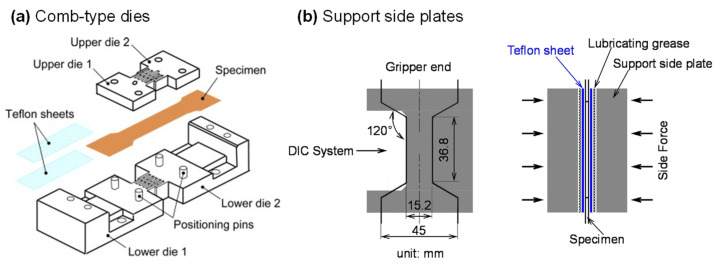
Experimental apparatus for UC tests of sheet metals: (**a**) configuration of the dies. Reprinted from Ref. [246] with permission. Copyright 2009, Elsevier; (**b**) the support side plates. Reprinted from Ref. [248] with permission. Copyright 2021, Elsevier.

Studies show that polycrystalline sheet metals such as magnesium and titanium alloys have obvious SD effects [243,244]. Thus, the measured stress–strain curves from the UT test cannot be used to describe the hardening behavior under UC. The thin sheet metal is prone to buckling when it is compressed, so the key to the mechanical characterization for measuring the UC stress–strain curve is the design of the anti-buckling fixture. Boger et al. [245] designed a hydraulic actuating fixture, which exerted pressure on the sample’s surface along the thickness direction through two parallel movable plates. A new method was proposed to correct the influence of friction force and sheet thickness stress. The non-contact laser extensometer was used to measure the strain distribution and history of the lateral surface of the sample. Kuwabara et al. [246] designed an interdigitated comb-like device as shown in Figure 17a to prevent the buckling of the specimen. A part of the test specimen surface was kept in normal contact with the support structure and the strain was measured using conventional strain gauges on the sample surface not supported by the comb device. Cao et al. [247] developed a wedge-shaped device to measure the stress–strain curve during compression using a geometry that ensures that the specimen is fully supported during testing. However, this design requires machining fins outside the specimen to enable strain measurements during UC loading. Hou et al. [248] measured the UC stress–strain curves of dual-phase steel sheets with a support fixture for suppressing buckling. The strain field was measured by the DIC method on one of the edge surfaces of the UC specimen (see Figure 17b). Hou et al. [249] further used this setup to determine the UC stress–strain curves of ultra-thin pure titanium bipolar plates based on a newly designed sandwich specimen. The above-mentioned UC anti-buckling fixture can be further utilized for the tension-compression cyclic loading experiment of sheet metals and realize the investigation of the Bauschinger effect, which will be detailed in Section 4.2.

#### 4.1.3. Simple Shear (SH)

SH experiments are often used to calibrate material parameters in advanced constitutive models or to determine the hardening curve of materials at large plastic strains [250]. In an SH experiment, the rectangular test area deforms into a parallelogram along its length when the width is fixed. Bouvier et al. [251] studied the stress and strain distribution of SH specimens and found that the uniformity of shear bands depends on the geometries of the SH specimen. They recommend a 10:1 ratio of shear area length to height to maximize the uniform region. A standardized shear test method ASTM-B831 [252] was established stipulating the shape and size of the SH specimen. Merklein and Biasutti [253] modified the ASTM standard specimen. Moreover, double bridge SH specimens were proposed [254,255,256]. There are boundaries in the gauge areas of the above SH specimens, so the materials near the boundary areas are always in a stress state of UT. There will be an interference of edge effect when calculating the shear stress. In order to avoid the influence of the edge effect, Marciniak [257] realized the measurement of SH loading of the copper plate for the first time by using in-plane torsion tests. Tekkaya et al. [258] used in-plane torsion experiments to determine flow stress curves for plates with equivalent plastic strain up to 1.0. Subsequently, in-plane torsion tests were further improved to measure the flow stress, kinematic hardening and fracture limit under SH of thin sheet metals [259,260,261].

#### 4.1.4. Hydraulic Bulging (HB)

**Figure 18:**
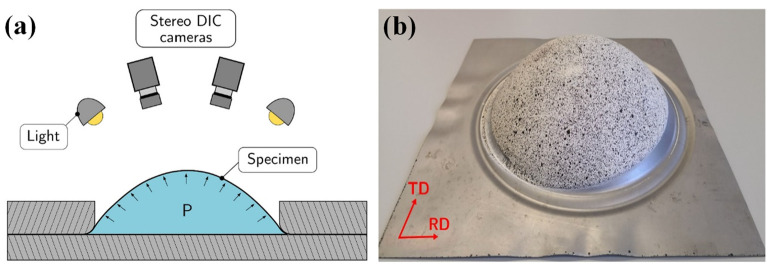
Hydraulic bulge test: (**a**) schematic of the experimental apparatus with DIC and (**b**) formed bulge specimen with DIC speckle pattern. Reprinted from Ref. [273], open access.

Lightweight sheet metals experience complex stress states including various biaxial deformation states, such as EBT and PS. Hydraulic bulging [262,263,264], pneumatic bulging [265] and bulging with viscous material as medium [266] experiments are advanced characterization methods to obtain stress–strain curves and the biaxial strain ratio in a large strain range under biaxial deformation [267]. An example of a bulge test set-up with a circular die and DIC measurement system is presented in Figure 18. The calculation method of stress–strain data in bulging experiments has undergone many years of development. Studies show that biaxial deformation in hydraulic bulging is sensitive to material anisotropy [262]. With the aid of DIC technology, Min et al. [268] proposed an accurate method for calculating stress and strain in circular hydraulic bulging experiments considering the anisotropic deformation of sheet materials. Despite some progress in the analytical measurement of stress–strain data, based on the membrane assumption, it is still necessary to use an experimental system with a ratio of die opening diameter to initial specimen thickness greater than 100 to ensure the validity of the computational method [264]. Lafilé et al. [269] proposed a new method to directly determine material behavior using DIC data on the outer surface of the specimen, which is suitable for hydraulic bulging experiments with small opening diameters of dies. A stress–strain curve close to the plane strain state can be obtained by hydraulic bulging with a reasonably designed elliptical die [270,271,272].

In addition, the tube expansion experiment can realize the plastic deformation of the material under various strain paths because it can control the axial tension or compression and radial bulging. Combined with the DIC method, the mechanical behavior under various strain paths can be accurately measured [274,275,276]. He et al. [274] developed a tube expansion testing system based on DIC feedback control, which realized the measurement of stress–strain curves in a large strain range under any biaxial loading paths in the first quadrant of the principal stress space. Tiji et al. [276] built a hydraulic bulging testing system based on finite element simulation and PID (Proportional Integral Derivative) control to achieve various linear strain paths.

The tube expansion experiment can realize the mechanical characterization of materials at large plastic deformation under various stress states. However, for sheet metals, it is necessary to prepare in advance closed tubular samples by methods such as laser welding. Sample preparation is difficult for high-strength materials and the influence of welding quality cannot be ignored. Moreover, the deformation of the specimen in the bulging experiment will be affected by the bending strain and the through-thickness stress, especially in the elastic or small plastic deformation stage. Therefore, bulging experiments are often inapplicable for advanced lightweight alloys, or accurate analytical methods are required to consider the effects of bending strain and through-thickness stress.

#### 4.1.5. Biaxial Tensile Testing with Cruciform Specimen (BTC)

Compared with experiments such as hydraulic bulging, BTC has some outstanding advantages: no bending strain, no thickness stress and no friction effect. In addition, the stress ratio can be controlled arbitrarily by the program of the BTC testing system. Therefore, BTC is widely used to study the hardening behavior under different BT conditions and to measure the yield locus of sheet metals [277]. In recent years, one of the main topics in the research on BTC is the design of cruciform specimens. The design of a cruciform specimen with uniform thickness mainly focuses on the geometrical design of the cruciform arms and the junction of the cruciform arms [278]. Kuwabara made outstanding contributions to the research on BTC [279,280,281]. The International Standards Organization established the ISO16842:2014 standard in 2014 [282]. ISO16842:2014 specifies the shape, geometric dimensions and processing method of the cruciform specimen with slits. However, when using ISO standard cruciform specimens, a challenging problem arises: the achievable plastic strain in the test area of the cruciform specimen is limited by the premature fracture of the arms of the cruciform specimen. There are two ways to increase the maximum plastic strain that can be achieved in the central region: (1) thinning the central test region; (2) strengthening the arms of the cruciform specimen. Liu et al. [283] designed a cruciform specimen with reduced thickness and a notch with a small central circular area. They conducted BT experiments combined with DIC to study the hardening behavior of AA5086 aluminium alloy under large strains. Recently, Zhang et al. [284] reviewed the development history of cruciform specimen designs, including 17 different geometries. Almost all of the above-mentioned cruciform specimens with thinned central test areas will produce fracture in the gauge area; that is, the premature fracture of the cruciform arms can be effectively avoided.

However, the thinning of the cruciform specimen in the central area generates some problems: (1) the thinning method by mechanical processing may introduce certain damage to the test material; (2) the thinning of the specimen often cannot ensure that the size and accuracy of the specimen thickness reduction in the test area meet the requirements of the sample design; (3) the thinned specimen in the central area must be suitable for determining the fracture limit of the thin-plate material. If it is used to characterize the hardening behavior of the thin-plate material, it needs to undergo analytical methods or combined with finite element simulation. The inverse process method should be used to calculate the stress–strain curves of the material under different biaxial loading conditions.

**Figure 19:**
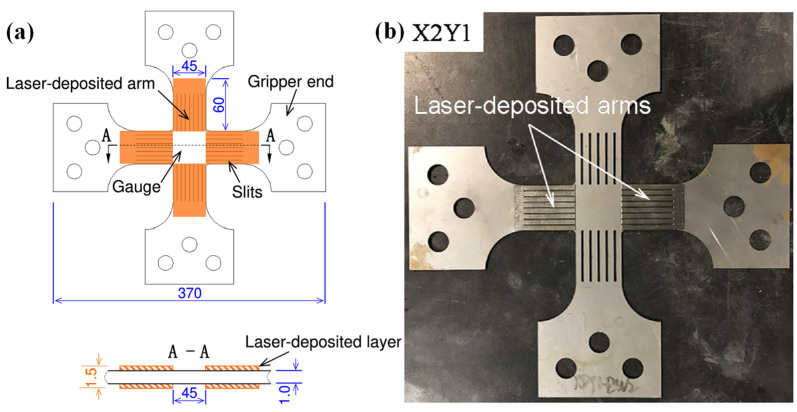
Cruciform specimen with arms reinforced by laser deposition: (**a**) dimensions and (**b**) a laser-deposited cruciform specimen. Reprinted from Ref. [248] with permission. Copyright 2021, Elsevier.

Another way to increase the maximum plastic strain achievable in the central region of the cruciform specimen is to strengthen the cruciform arms. There are several innovative ways to strengthen the outer region to increase the plastic deformation of the cruciform specimens [285,286,287]. The achievable plastic strain range in the gauge area of a cruciform specimen can be significantly increased using novel specimens by Hou et al. [248] with laser deposition on the arms (as shown in Figure 19) in comparison with ISO Standard specimens for three dual-phase steels with strength grades from 590 to 980 MPa. Evolving yield behavior can be experimentally measured up to large plastic strains, e.g., ~0.11 for DP590, ~0.07 for DP780 and ~0.05 for DP980.

### 4.2. Non-Proportional Loadings

Sheet metals are often subjected to complex strain histories, e.g., SPCs, during stamping of automotive components, for instance, sheets undergo a bending-reverse-bending deformation process when flowing through a draw bead or die fillet. Therefore, the mechanical characterization of materials under non-proportional loadings is of great significance in studying Bauschinger effect related hardening behaviors.

#### 4.2.1. Uniaxial Tension-Uniaxial Compression (TC)

The TC cyclic loading experiment is the most widely used experimental method to study the Bauschinger effect of sheet metals. The key to developing the mechanical characterization for measuring the hardening curve of the sheet metals under TC is the same as that of the UC tests, which require the design of the anti-buckling fixture. The measured stress–strain curves under TC loadings are used to characterize the Bauschinger effect and to calibrate and validate the kinematic or distortional hardening models [21,170,179,245,288,289,290,291,292].

#### 4.2.2. Shear-Reverse Shear (SRS)

The SRS test is an important experimental method to study the Bauschinger effect under the load-reverse load state [182,185,293,294]. Bouvier et al. [251] used the optimally designed shear testing fixture to achieve uniform distribution of stress and strain in the shear region of the SH specimen and to obtain stress–strain curves under SRS by changing the direction of loading. Yin et al. [260] developed an in-plane torsion experimental system that combines shear-reverse shear loading with the DIC technique. The advantage of this experiment is that only one experimental test is performed on one sample and the determination of stress–strain curves under different pre-strains can be realized. Zou et al. [139] designed a lateral support fixture to prevent the shear specimen from buckling and measured the stress during SRS experiments of two advanced high-strength steels DP980 and QP980.

#### 4.2.3. Bending-Reverse Bending (BRB)

The BRB test can also be used to determine the Bauschinger effect in thin sheet materials. The inner/outer layer material undergoes a compression-tension/tension-compression deformation history during the BRB test. Yoshida et al. [295] designed a cyclic bending experimental setup to perform BRB experiments on three thin sheet metals and proposed an advanced parameter optimization technique based on the measured bending moment-curvature curves. Beginning with the work of Yoshida [296], the determination of the cyclic stress–strain response from cyclic bending experiments using the inverse method has gained some popularity [296,297]. Zang et al. [298] proposed an experimental method to characterize the Bauschinger effect of materials by using the three-point bending springback test of the specimen after pre-tension deformation. For pre-tensioned specimens, the subsequent three-point bending experiment will cause the inner layer of the sheet to undergo a reverse loading of first tension and then compression, so it can be used to study the Bauschinger effect of the material. More recently, this method was adopted by Choi et al. [299] to calibrate the parameters of the HAH model for ultrathin sheet metals.

#### 4.2.4. Non-Reverse Strain Path Changes

**Figure 20:**
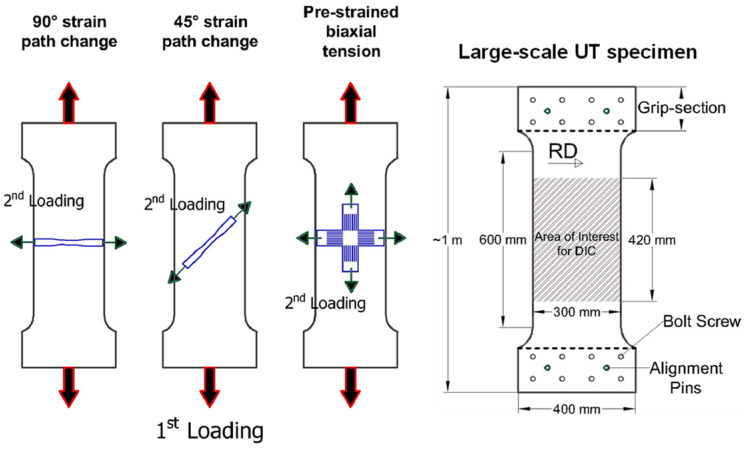
Schematic illustration of the selection of standard specimens from the uniform region of the large-scale specimen. Reprinted from Ref. [302] with permission. Copyright 2018, Elsevier.

There are some experimental characterization methods for non-reverse SPCs. Barlat and co-workers made outstanding contributions to the characterization of the mechanical properties of large specimens with UT preloading and related research on the Bauschinger effect [300,301,302,303,304]. Zaman et al. [302] cut sub-size UT and cruciform specimens from the pre-deformed specimens for secondary deformation (Figure 20).

**Figure 21:**
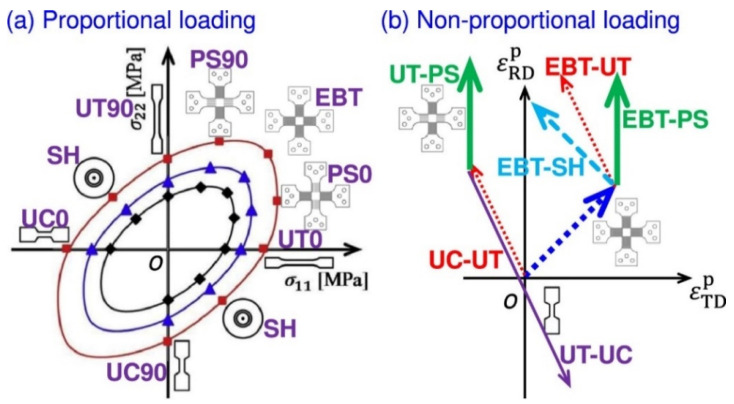
Mechanical characterization approaches used to investigate the evolving yield surfaces under (**a**) proportional loading and (**b**) non-proportional loading. Reprinted from Ref. [184] with permission. Copyright 2022, Elsevier.

Wi et al. [303] designed a smaller cruciform specimen to characterize the hardening behavior in the BT stress state. The stress–strain curves under various SPCs were measured, providing experimental data for calibrating and validating advanced constitutive models. Using the commonly-used cruciform specimens, it is difficult to measure the strain hardening curves under large plastic strains [282]. For advanced high-strength materials, only the initial yield stress could be measured and the subsequent yield surface evolution of the material could not be obtained. Recently, the complex anisotropic hardening behavior of a Q&P steel sheet with a strength of 980 MPa (QP980) subject to biaxial proportional and non-proportional loadings was investigated through advanced mechanical characterization, as shown in Figure 21. The biaxial SPCs, e.g., UT followed by PS, EBT followed by PS, UT and SH, were successfully characterized by the BT tests with arm-reinforced cruciform specimens.

In summary, advanced lightweight alloys exhibit complex mechanical properties, which puts forward new requirements for the advanced constitutive models and characterization methods under complex loading conditions. Complex loading conditions are mostly limited to reverse loadings such as TC, SRS and BRB. Hence, novel mechanical characterization methods under SPCs are required for the engineering application of advanced lightweight alloys.

### 4.3. Inverse Engineering

Advances in optical and numerical techniques have led to developing a new generation of characterization methods such as inverse engineering to understand the complex deformation behavior of materials or components. These techniques, including DIC, finite element model updating (FEMU) and virtual fields method (VFM), enable new approaches to generate more knowledge about plastic deformation behavior. Recent reviews of the research on the optimization and inverse analysis of metal forming and heterogeneous mechanical tests for the identification of material parameters can be found in Andrade-Campos et al. [305] and Pierron and Grédiac [306]. FEMU compares measurable variables to obtain the cost function: (1) local observations, such as the strains and (2) global observations, such as the load, while VFM uses a balance equation between the external and internal virtual work to determine the parameters.

As mentioned in Section 4.1, the measured stress–strain curve from the UT test is limited due to the localized necking of the specimens; hence, inverse engineering can be used to determine the post-necking responses. Approaches for obtaining stress–strain curves at large strains (or strain at post-necking process) by coupling experiments (or load-displacement curve) with finite element analysis have been developed [307,308]. Pham et al. [309] coupled the inverse finite element analysis and the curve-fitting method to identify the post-necking stress–strain curves. The high flexibility was highlighted in the selected hardening laws.

Another significant application of inverse engineering is identifying an anisotropic yield function. Lou et al. [310] identified the optimized parameters for the proposed yield function and the Swift-Voce hardening law. The numerical simulation errors were calculated for mechanical tests with the dog-bone specimen, the central hole specimen, the notched specimen and the in-plane shear specimen by comparison of predicted load-stroke curves with experiments. The downhill simplex algorithm was selected as the optimization algorithm. Zhang et al. [311] compared the analytical computation and inverse engineering approach under different stress states (simple shear, uniaxial tension, plane strain tension and equi-biaxial tension) of the AA5182-O sheet. The evaluation showed that the inverse engineering approach could effectively characterize the strain hardening curve up to large plastic strains, especially for tests with inhomogeneous deformation.

**Figure 22:**
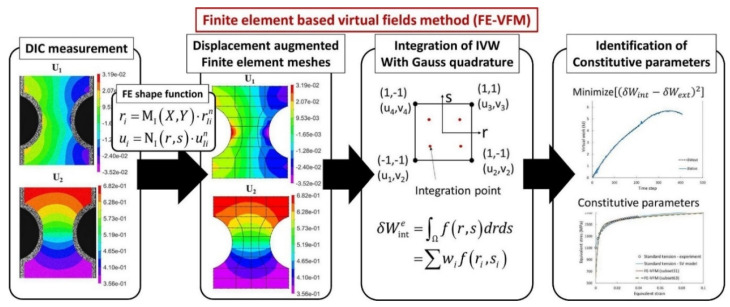
Finite element-based virtual fields method with pseudo-real deformation fields for identifying constitutive parameters. Reprinted from Ref. [314] with permission. Copyright 2021, Elsevier.

**Figure 23:**
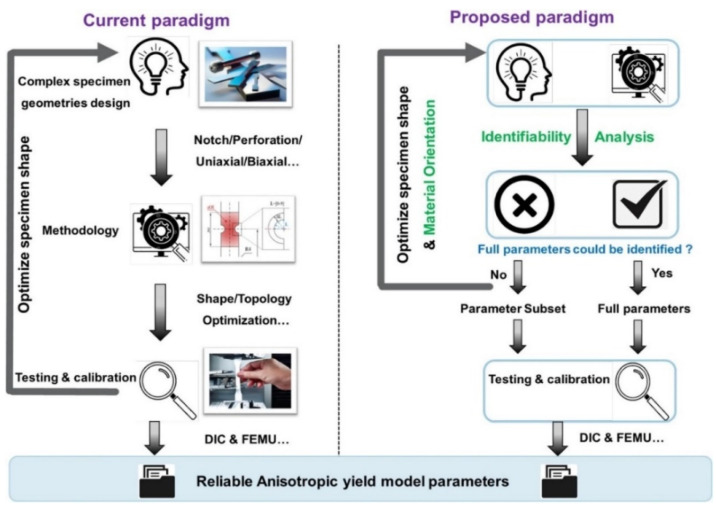
Comparison of current and proposed paradigm for non-conventional test design using specimens of complex geometries and parameters identification. Reprinted from Ref. [317], open access.

Inverse engineering with full-field measurements, e.g., VFM, is increasingly and widely employed in identifying constitutive parameters of anisotropic plasticity models. Grédiac and Pierron [312] presented the first attempt to apply VFM to identify elasto-plastic constitutive parameters, where a very simple Prandtl-Reuss law was considered. Rossi et al. [313] described an application of VFM to large-strain anisotropic plasticity. Full-field data of notched specimens could be processed with low computational times to identify the constitutive parameters of plasticity models. Recently, Kim et al. [314] proposed a new VFM based on real nodal displacements to improve the accuracy of VFM at large plastic deformation, as shown in Figure 22. Using cruciform specimen, Martins et al. [315] explored a potential test to simultaneously identify the parameters that govern an anisotropic yield criterion and a hardening law using the virtual fields. Moreover, the different complex geometries were used to enhance and further increase the robustness of the used VFM [316,317], as shown in Figure 23. A comparative study of four identification strategies based on full-field measurements was conducted by Martins et al. [318] and the results showed that VFM could be a perfect candidate for achieving a reasonable balance between the quality of the identification procedure and computational cost.

## 5. Evaluation and Modelling of Forming Limit

**Figure 24:**
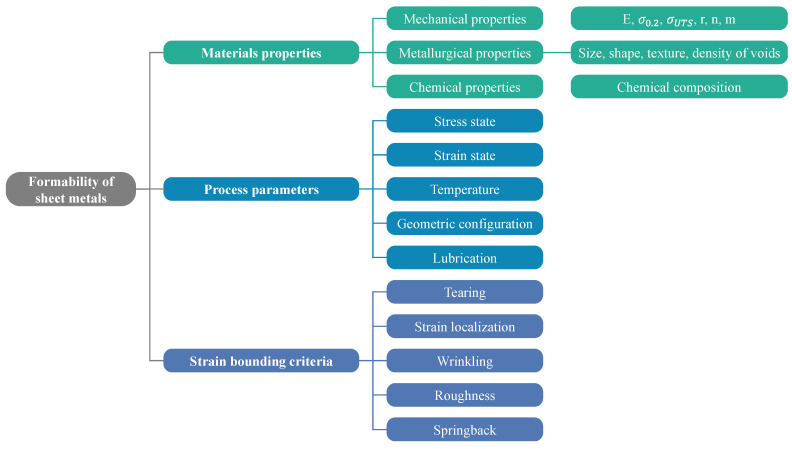
Factors affecting the formability of sheet metals.

The formability of sheet metal is defined as its ability to resist the plastic deformation before the onset of fracture. It can be used to describe the flow and anisotropic behaviors, plastic anisotropy and the forming limit diagram/curve (FLD/FLC) which is characterized through determining two principal strains at fracture called major strain (*ɛ*_1_) and minor strain (*ɛ*_2_) [319]. Unveiling the formability of sheet metals is challenging because it depends on the many factors in Figure 24.

**Figure 25:**
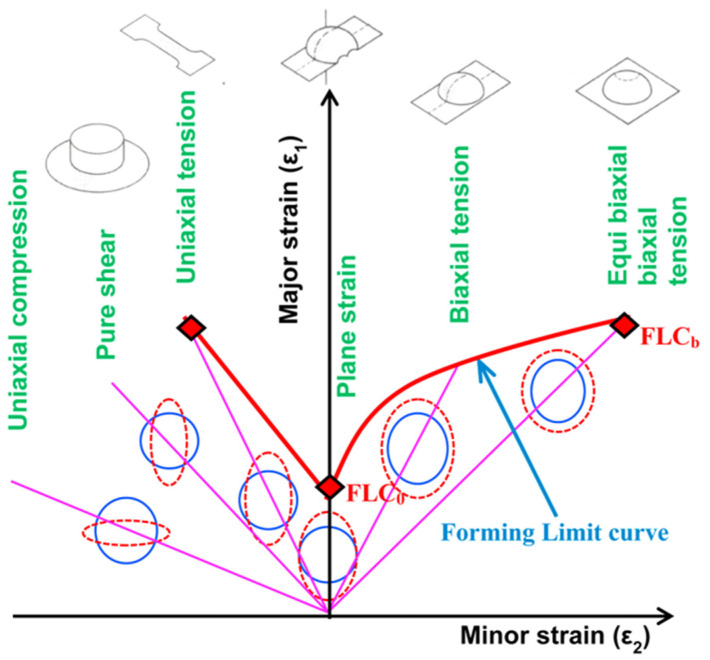
A schematic description of FLD and other failure limits. Reprinted from Ref. [325] with permission. Copyright 2016, Elsevier.

FLD is considered as a critical tool used for evaluating sheet metal’s formability. Keeler proposed the negative region of the minor strain of FLD, then Goodwin [320] extended this by proposing the positive region of the minor strain. Extensive and standardized experimental techniques were used to determine the FLDs of sheet metals under different forming conditions such as forming temperatures and out-of-plane (Nakajima test) and in-plane (Marciniak test) formability testing [321,322]. Furthermore, the machines used for planar-biaxial tensile testing can also be utilized for determining FLDs of sheet metals because of their ability to control strain path precisely and avoid the impact of friction [323,324]. The details of FLD are depicted in Figure 25 [325]. As depicted, FLC is the crucial feature of FLD, where it can describe the strains limit at the necking onset. FLC is defined by the plotted major strain ($\varepsilon_{1}$) and minor strain ($\varepsilon_{2})$ which were determined from the formability testing under different strain paths from uniaxial to biaxial tensions. FLC highly depends on strain rates and forming temperatures and its shape depends on the strain path. Besides, the microstructure can seriously affect the formability of lightweight materials. Zecevic et al. [326] found that the continuous-bending-under-tension process postponed the onset of necking and significantly increased the percentage elongation at failure of AA6022-T4 sheets. A similar phenomenon regarding formability enhancement was also reported in other lightweight metallic materials [327,328,329], where the underlying mechanisms behind the enhancement of formability were discussed in detail. Regarding the formability of lightweight sheet metals, the regions under and above the FLC describe the safe forming and instability or necking regions, respectively [330,331].

**Figure 26:**
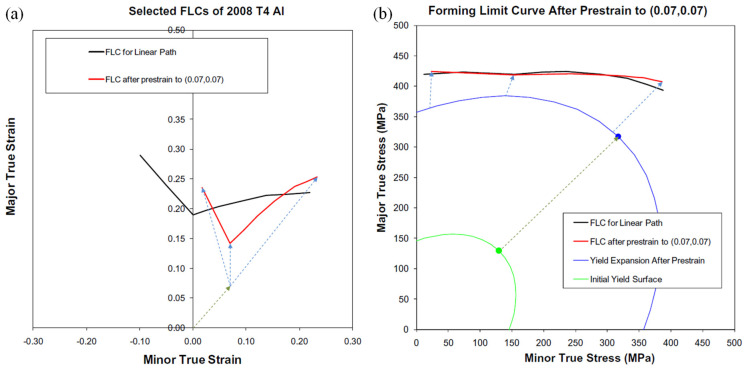
The transformation from an FLD to an FLSD. (**a**) FLD which contains two FLCs including one FLC with pre-strain; (**b**) transformed FLCs in the FLSD. Reprinted from Ref. [335] with permission. Copyright 2012, Elsevier.

The main issue of a determined FLD is its validity only for a process where the straining path is linear and the loading is proportional [332]. Since strain paths are commonly not linear in the multistage forming processes, the influence of the non-proportional strain paths restricts the adequacy of the FLDs for evaluating the formability of sheet metal. To address this issue, Kleemola and Pelkkikangas [333] and Arrioux et al. [334] offered FLSD, which does not depend on strain paths. The FLCs of the FLSD cannot be determined straightforwardly from experimentation, but they are represented through major principal stress and minor principal stress as coordinates. Thus, Stoughton and Yoon [335] proposed a new model-based on Hill’s yield criterion and hardening law to transfer the FLDs of metallic materials into FLSDs, as shown in Figure 26.

**Figure 27:**
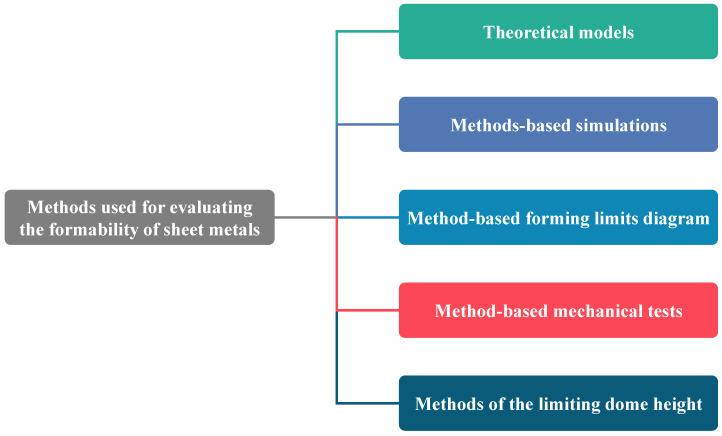
Methods used to evaluate the formability of sheet metals.

The experimental techniques to determine FLDs of sheet metals are costly and time-consuming notably testing at elevated temperatures [336]. Thus, many researchers have and developed different numerical techniques and theoretical models which can be implemented easily in numerical simulation software for predicting and analyzing sheet metal’s formability, as summarized in Figure 27.

**Figure 28:**
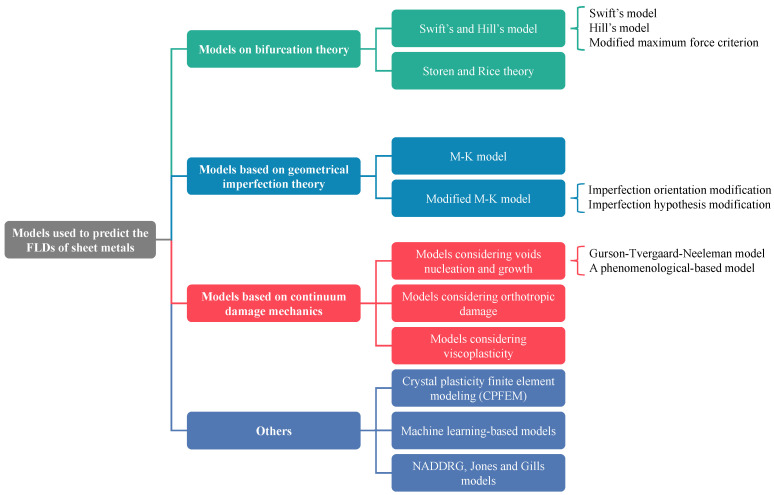
Theoretical and numerical models used for formability prediction.

Zhang et al. [337] discussed in their study the primary empirical and theoretical models used for evaluating and predicting the formability of sheet metals and categorized them into bifurcation theory-based models, geometrical imperfection theory-based models, continuum damage mechanics (CDM) models and other models based on necking or fracture criteria, as depicted in Figure 28. The details of these models are discussed briefly in Section 5.3. Afterwards, Stoughton and Zhu [332] proposed a theoretical FLD model-based strain and explained its relevance with FLSD. Then, many reviews were performed by Aretz [338], Stoughton and Zhu [332], Hosford and Duncan [339] and Banabic et al. [340] to discuss the development and the progress of different theoretical models such as Hill’s, Swift’s and M-K models, for several metallic materials, strain paths and process parameters through coupling various hardening laws and yield criteria.

### 5.1. Measurement of Surface Strains in Sheet Metal Forming

**Figure 29:**
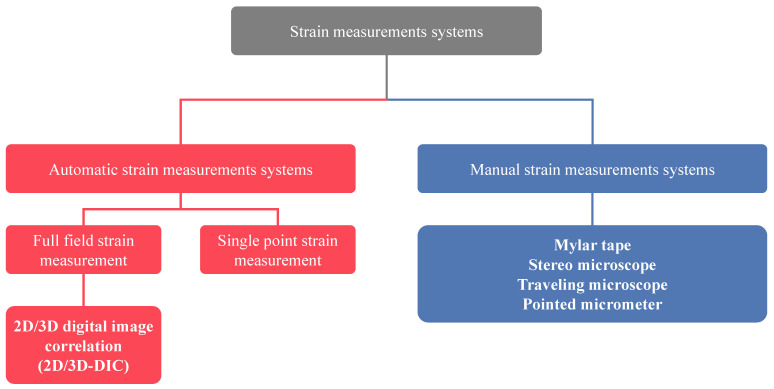
Classification of automatic strain measurement system.

Measuring surface strains is one of the essential requirements for analyzing and evaluating the formability of sheet metals. Therefore, it is crucial to complete this stage because most of metallic sheets used for forming processes have thicknesses less than 1.5 mm, which may affect the strain measurements. Many techniques (manual and automatic) are used for strain measurements, as summarized in Figure 29 [341].

**Figure 30:**
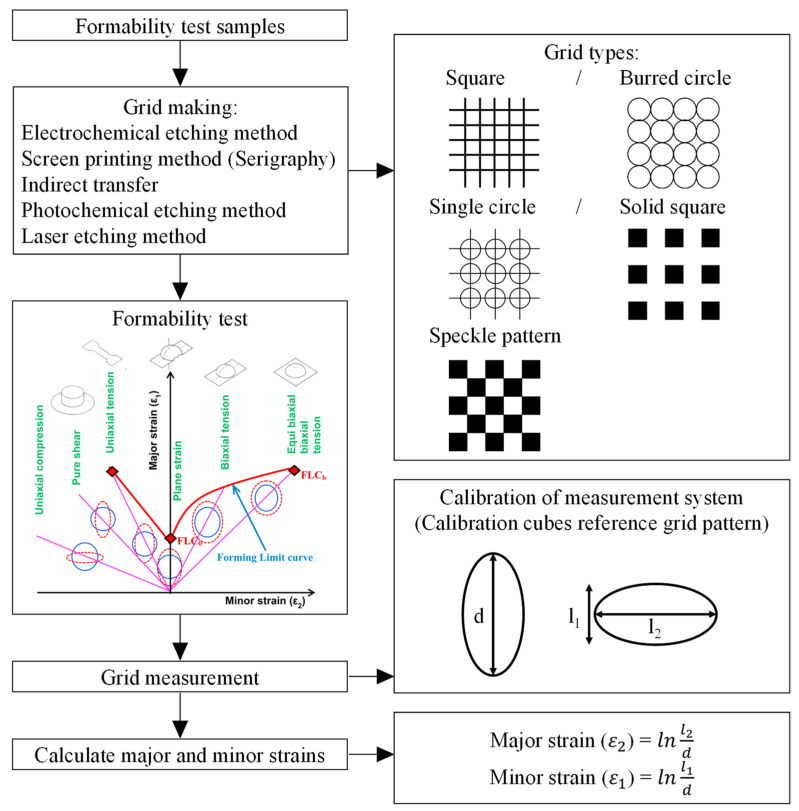
Detailed procedures of measuring major and minor strains manually. Reprinted from Ref. [325] with permission. Copyright 2016, Elsevier.

The manual techniques include a stereo microscope, travelling microscopes and mylar tapes. Bandyopadhyay et al. [342] proposed a manual technique for strain measurement and used these results and their theoretical model to build FLSD to investigate the formability of TWB. Prasad et al. [343] used a stereo microscope technique for measurement of strains and studying the formability of Inconel 718. Nevertheless, their techniques are less accurate and time-consuming than automatic techniques. Figure 30 describes the detailed procedures for measuring major and minor strains manually.

The available automatic strain measurements techniques are for full-field, such as DIC, and single-point strain analyses, as summarized in Figure 29. In GPA, after forming, the deformed grids were captured by a camera, and software was used to fit the ellipse to deformed grids and determine the major and minor the deformed grids strains. On the other hand, the full-field technique commonly uses the principles of DIC to measure strain automatically [344]. This technique assesses deformation and strains with the assistance of a minimum of two images with a mutual geometrical relation. Omar et al. [344] used offline DIC software ARGUS which was proposed via GOM for measuring strains to study the forming limits of welded steel tubes manufactured by hydroforming. Bhargava et al. [345] used an online ARAMIS system to measure strains and construct the FLDs for AHSS sheets. Sutton et al. [346] offered a review discussing DIC’s concept, theory and applications for strain measurements. Khoo et al. [347] explained the concept, theory and applications of the 2D-DIC system. McCormick et al. [348] presented the benefits and uses of DIC to analyze the crack propagations in real scenarios using a low-cost experimental setup.

### 5.2. Experimental Determination of Forming Limit Diagram (FLD)

The classical stretching tool used to assess sheet metal formability is a punch with a diameter of 20 ± 0.05 developed via Erichsen [349]. Then Olsen [350] modified the test method proposed by Erichsen, but using a different tool size. Afterwards, Hecker [351] introduced a new test method based on the concept of the tests developed by Olsen and Erichsen and used a punch with a bigger diameter of 50 mm to overcome the limitations of the aforementioned tests which were caused by using punches with small diameters. Kotkunde et al. [352] also used Hecker’s test in their study and compared the test results predicted from his theoretical model. Jovignot [353] developed a new hydraulic bulging testing machine used notably for equi-biaxial strain path. The formability of sheet metals is usually assessed experimentally by Nakajima test (out-of-plane) [354] or Marciniak test (in-plane) [321]. The test setups and the assessment techniques of Nakajima and Marciniak tests to determine FLDs for sheet metals are standardized via ASTM E2218-15 [355] and ISO 12004–2 [356]. In the Nakajima test, known as limiting dome height (LDH), a hemispherical punch is used; however, a flat bottom cylindrical punch is used for the Marciniak test. Many investigations have been performed to evaluate the formability of sheet metals apart from the standard procedures by using other samples and punch sizes in stretching operations to plot the FLD, where the standard diameters of the hemispherical punch and blank which should be used are 101.4 and 177.8 mm, respectively [357,358,359]. Several methods were proposed to determine the onset of localized necking in order to improve the accuracy of FLD [360,361]. The methods can be classified into three categories: (1) spatial methods, e.g., from ISO 12004-2 [356] and Zhang et al. [362]; (2) temporal methods, from Volk and Hora [363], Merklein et al. [364] and Hotz et al. [365]; (3) Spatio-temporal methods, from Li et al. [366], Wang et al. [367], Martínez-Donaire et al. [368] and Min et al. [369,370].

Some vital parameters affect the formability test and stretching operation, such as the size of punch and die, blank holding pressure (approximately 2% of the material’s yield strength) and draw beads, which are provided for resisting the material flows from the flange region. The punch size is crucial to determine accurate FLD, as mentioned by Basak et al. [371]. They used a sub-size punch in their study to reveal the impact on the accuracy of determining the FLD of sheet metals. They mentioned that sub-size punches are responsible for inducing bending strains in the sheet’s outer surfaces which could be overcome by subtracting the bending strains from both major and minor strains. On the other hand, these experimental techniques are time-consuming, costly and complicated and require many specimens for testing under different strain paths. To address the aforementioned issues and overcome the limitations of the experimental procedure, many theoretical and empirical models have been proposed to predict the FLDs of sheet metals. Yield criteria and the hardening model are the key parameters which should be considered to determine the limit strains using theoretical models.

### 5.3. Determination of FLD via Modelling Techniques

#### 5.3.1. Models Based on Bifurcation Theory

In 1952, Hill [372] and Swift [373] proposed classical models for predicting localized (through-thickness direction) and diffuse necking, respectively, and they assumed that the sheet metal is homogeneous. Then, Hill’s model was developed by Aretz [374], where it was assumed that the localized necking occurred once the large forces per unit widths reached to the critical values, not maximum values. Then, Sing and Rao [375] and Chung et al. [376] developed Hill’s criterion to determine the forming limits of several steel sheets such as SS4011, DP600 and TWIP940. On the other hand, they can only predict the LHS of the FLDs of sheet metals because of the zero-extension hypothesis. Thus, Swift [373] introduced his model, known as maximum force criterion (MFC), which is based on diffuse necking for the sheet metals deformed biaxially. Swift [373] assumed in his model that the diffuse necking occurring and the formability limit on strains can be determined once the loading reaches to maximum value. On the other hand, the obtained results commonly underestimate the formability limits strains detected from experimentation [375]. Hora et al. [377,378] considered the impact strain state and modified Swift’s model, known as MMFC. Then, Hora et al. [379] performed further modifications to their model and considered the influence of forming temperatures. Storen and Rice [380] introduced a new model (S-R model) for predicting the formability limits strains at the necking onset by assuming a vertex on subsequent yield locus. Then, Hashiguchi and Protasov [381] developed an S-R model to determine the FLDs of several elastoplastic materials under different working conditions. Afterwards, Zhu et al. [382] considered the moment equilibrium and modified the S-R model and Min et al. [383] successfully determined the FLD of 22MnB5 sheet metal at elevated temperatures by coupling the Logan-Hosford criterion with the S-R model.

#### 5.3.2. Models Based on Geometrical Imperfection Theory

**Figure 31:**
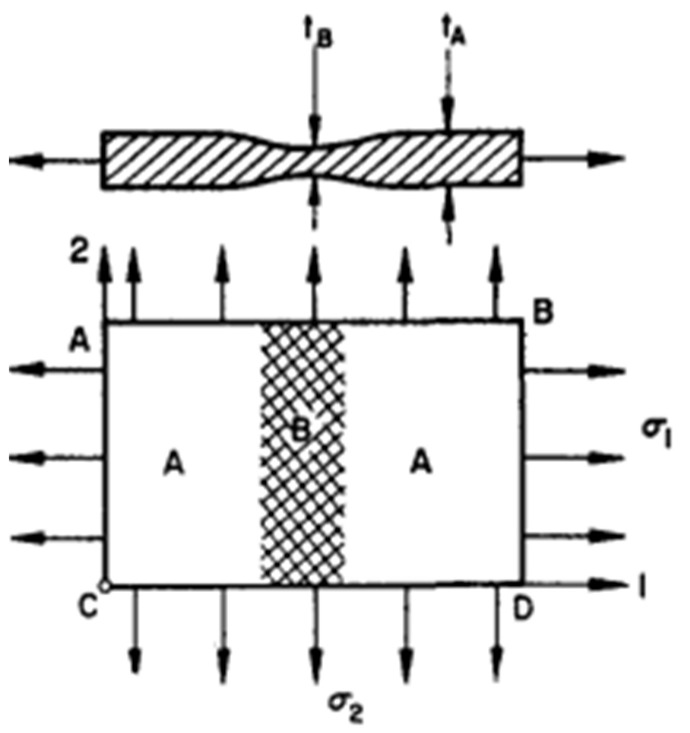
A schematic description of the pre-existing groove supposed in the M-K model before the deformation. Reprinted from Ref. [321] with permission. Copyright 1967, Elsevier.

Marciniak and Kuczynski [321] proposed a new model (M-K model) based on the geometrical imperfections on the sheets before the deformation to determine the FLD of sheet metals, where the imperfections are assumed to be normal to the major principal stresses, as shown in Figure 31. Then, Hutchinson et al. modified the M-K model [384,385] to predict the FLD of sheet metals by considering an arbitrary angle between the minor principal stress and imperfection. The formability limit strains determined via the M-K model are sensitive to the size of the geometrical imperfection, which is impractical for the applications of formability limit strains predictions [386]. The formability limits strains predicted via the M-K model are commonly overestimated, notably at a high strain ratio [387].

**Figure 32:**
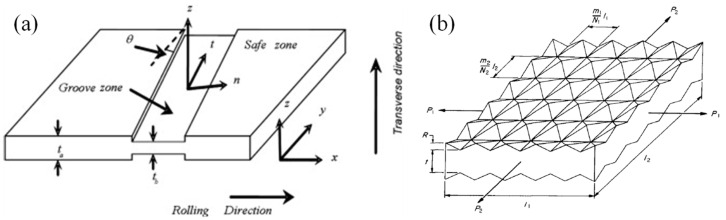
Schematic descriptions of the (**a**) pre-existing groove. Reprinted from Ref. [392] with permission. Copyright 2009, Elsevier; and (**b**) surface roughness considered in the modified M-K model. Reprinted from Ref. [390] with permission. Copyright 1977, Elsevier.

To address the aforementioned issue, the M-K model was modified via considering the voids growth [388,389] and the roughness surface [390] in the imperfection hypothesis, as depicted in Figure 32. As shown in Figure 32a, based on the zero extension hypothesis proposed by Hill, the imperfection groove was modified in the M-K model. inclined with an angle θ to the minor principal stress [384,385,391,392], as shown in Figure 32b. For instance, Parmar et al. [390] considered the surface roughness of sheet metals in the M-K model and determined the formability limit strains of Al alloys sheets utilizing both modified M-K and Swift’s models. They first predicted the sheet’s instability via Swift’s model; afterwards, they used the modified M-K model to determine the formability limit strains. Thereafter, Bong et al. [393] used the same models (i.e., Swift’s model for sheet instability and M-K model for formability limits) to determine the FLDs for stainless steel sheets and verified their results with those obtained from experimentation. Chan et al. [394], Hashemi et al. [392], Panich et al. [395] and Abedrabbo et al. [396] also modified the M-K model in their studies and predicted the FLDs of different metallic materials such as Al and steel alloys. Needleman and Triantafyllidis [388] and Melander [389] considered the impact of void growth in their modified M-K models and determined the forming limit strains in their investigations.

#### 5.3.3. Models Based on Continuum Damage Mechanics (CDM)

The ductile fracture process can be classified into three stages, micro-voids nucleation, growth and coalescence [397]. GTN is one of the models based on these processes and has been used to determine the formability limit strains of metallic sheets [398]. Lin et al. and Shao et al. [399,400] proposed new stress-based CDM theories for predicting the FLDs of Al alloy sheets at a wide range of elevated temperatures. Brunet and Morestin [401] modified the GTN model based on Hill’s anisotropic yield surface and the necking criterion of Swift’s model to determine the formability limits of Al and Ti alloy sheets. Chen and Dong [402] modified the GTN model, using Hill’s quadratic stress instead of von Mises stress to determine the FLDS of AA6111-T4 sheets. Chow et al. [403,404] proposed anisotropic damage models for predicting the FLDs of AA6111-T4 sheets at different forming conditions. Then, they developed their model and coupled it with Hill’s 48 yield criterion and mixed isotropic-kinematic hardening models to determine the FLD of AA6111-T4 at complex strain paths [405].

#### 5.3.4. Alternative Models

In 1975, a new empirical model was proposed by Keeler and Brazier [406] to determine the major principal strain at plane state. They assumed in their study that FLD consisted of two lines inclined on the major principal strain with an angle of 20° for the RHS and 45° for the LHS in the FLD. Then, Bleck et al. [407] utilized this model for predicting the formability limits for IF and DP steel sheets, because it is suitable only for ultra-deep drawable steels. Slota and Spisak [408] obtained the same conclusions when they used this model in their study to determine the FLDs of various steels. Afterwards, Djavanroodi and Derogar [409] utilized Keeler’s model for predicting the FLDs of both Al6016-T6 and Ti6Al4V sheets; however, the model tended to overestimate the formability limits. Jones and Gillis [410] introduced a new model (J-G model) to determine formability limit strains at the beginning of necking. Still, they obtained the formability limits in RHS of the FLD only because of their assumption that the neck direction is normal to major principal stress. Then, Choi et al. [411] modified the J-G model and determined the FLDs for different Al alloys sheets. Compared with the experimental results, the developed J-G model can successfully determine the FLDs for AA2036-T4 and AA1100-H19 alloys but not for AA3003-O alloy.

**Figure 33:**
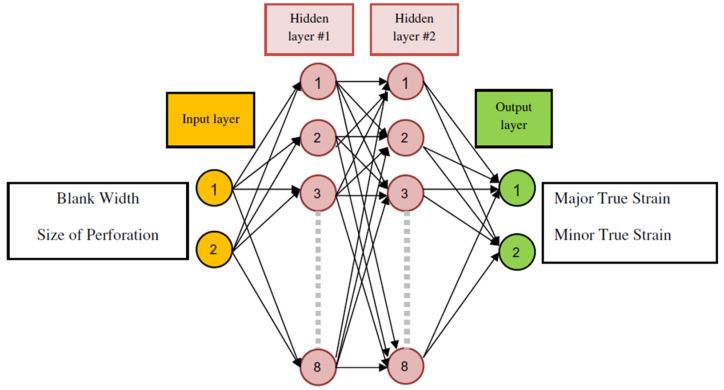
A schematic description of ANN-based model. Reprinted from Ref. [412] with permission. Copyright 2010, Elsevier.

Artificial neural network (ANN) has also been applied to predict FLDs for a perforated sheet with different geometrical features which are the input of this model, while limit strains are the output, as depicted in Figure 33 [412]. It is required in the ANN model to train huge experimental data; afterwards, the limit strains can be determined at other forming conditions. For instance, Elangovan et al. [412] and Kotkunde et al. [413] developed their own ANN-based models for predicting the FLDs of pure Al alloys and Ti-6Al-4V alloy, respectively.

**Figure 34:**
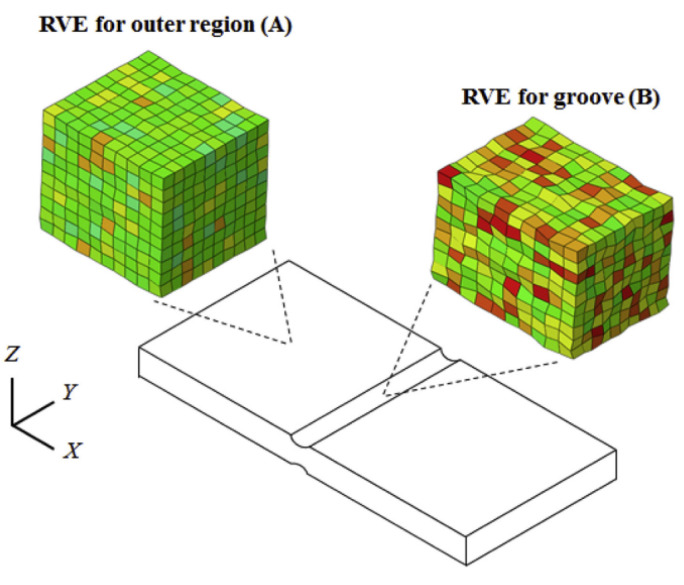
A schematic description of a multiscale approach that coupling the M-K model and CPFEM. Reprinted from Ref. [420] with permission. Copyright 2017, Elsevier.

**Figure 35:**
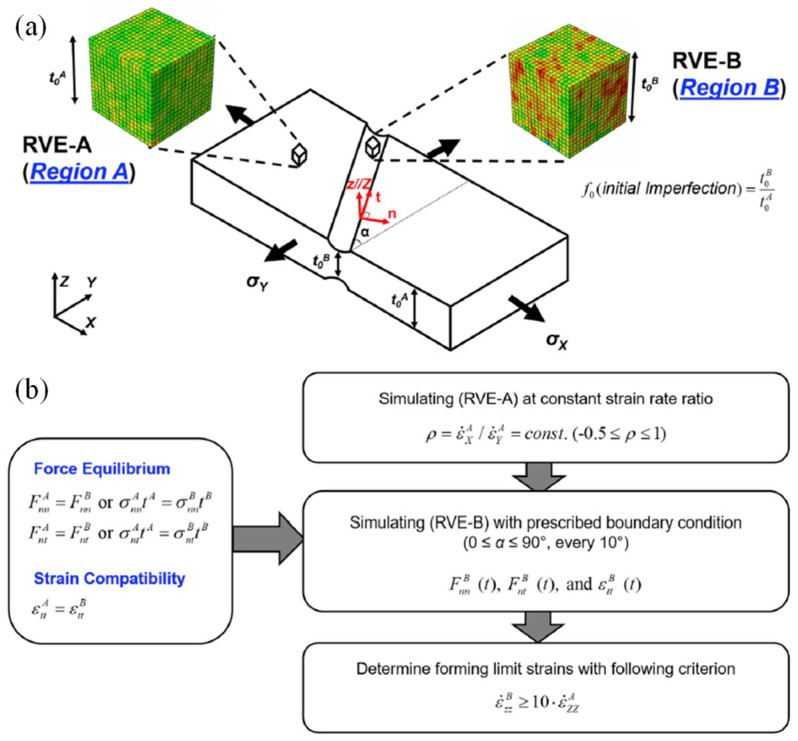
A schematic description of the novel multiscale approach proposed by Bong et al. [437] to predict the FLDs of AZ31 and ZE10 Mg sheets: (**a**) schematic of the proposed hybrid approach; (**b**) flowchart for the multiscale FLD prediction. Reprinted from Ref. [437] with permission. Copyright 2020, Elsevier.

As mentioned previously, most theoretical models used to predict the FLD of metallic materials are based on the M-K model, where initial linear imperfections are typically assumed on the sheet’s surface. Then, many investigations were performed to modify the M-K model and improve its prediction capability by considering other microstructure inhomogeneity. For instance, Wu et al. [414], Savoie et al. [415], McGinty et al. [416], Inal et al. [417], Neil and Agnew [418], Wang et al. [419] and Kim et al. [420] considered sheet texture to improve the modified M-K model. Besides, Ragab and Saleh [421] and Bong et al. [393,422] considered void density and surface roughness in their investigations. Karafillis and Boyce [423] and Barlat et al. [123,424] combined the M–K model with advanced models especially yield function, to consider the plastic anisotropy of sheet metals before and after deformation. Furthermore, Banabic et al. [340], Bong et al. [425] and Panich et al. [395] proposed several M-K-based techniques to predict the FLD of sheet metals with acceptable precision at room and elevated temperatures. Nevertheless, it is still challenging to consider plastic anisotropy due to the change in the shape of the yield surface during deformation. The considerations of the plastic anisotropy in Mg alloys is more important than those of FCC and BCC sheet metals because of their low crystal symmetry and limited slip activities [426]. CP modelling can track the plastic anisotropy of sheet metals by considering textures changes during non-proportional loading [238]. Thus, researchers employed CP modelling to predict the FLDs of such sheet metals [414,415,416,417,418,419,420,427,428,429,430,431] by coupling the M-K model with crystal plasticity. Their new framework was based on the RVE, representing a single material point on the metallic sheet. Tadano et al. [432] used the homogenization framework developed by Guedes and Kikuchi [433] and coupled it with the M-K model to determine the FLDs of FCC sheet metals. Besides, they introduced a novel technique to predict the localization onset via simulating the RVEs for two material points only. Hajian et al. [434,435] combined the M–K model with their new 2D-CPFE model. They assumed initial imperfections oriented along different directions and simulated the forming limits criterion. The above-mentioned models [429,432,433,434,435] could not precisely solve the issue of heterogeneity of stresses and strains in the thickness direction. Thus, researchers proposed the 3D-CPFE model with multi-grains in the thickness direction and introduced multiscale frameworks-based CP modelling to predict FLDs accurately. However, there are limitations in the computational time required for the frameworks proposed by Srivastava et al. [427] and Mohammed et al. [436]. Kim et al., [420] tried to address the aforementioned issue by proposing a computationally effective multiscale approach by combining the M-K model with their CPFE model to determine the forming limit strains of BCC sheet using 2 RVEs for imperfection and uniform areas, as depicted in Figure 34. As depicted in Figure 35, Bong et al. [437] proposed a novel framework that combined their modified CPFE model and M-K model, which successfully predicted the FLDS of AZ31 and ZE10 Mg sheets.

**Figure 36:**
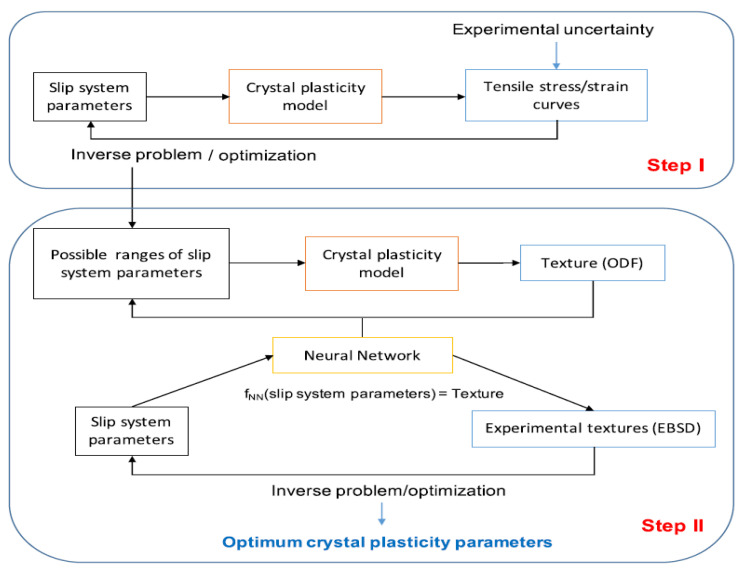
A schematic description of the novel multiscale approach proposed by Acar [451]. Reprinted from Ref. [451] with permission. Copyright 1963, AIAA.

**Figure 37:**
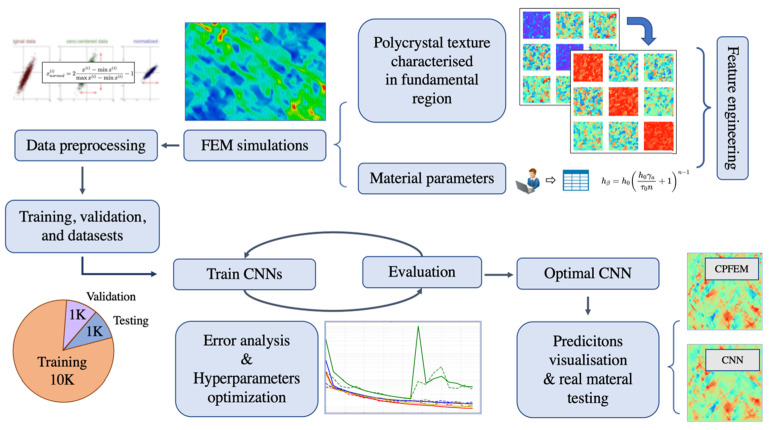
A schematic description of the novel framework proposed by Ibragimova et al. [452] to predict the FLDs of AZ31 and ZE10 Mg sheets. Reprinted from Ref. [452] with permission. Copyright 2022, Elsevier.

Although CP has been regarded as an outstanding modelling tool, it requires higher computational time for its broad applications. Thus, several techniques such as a spectral database technique [438,439,440], wavelet transformations-based algorithm [441,442], graphics processing unit (GPUs)-acceleration of CP [443,444] and parallel computing [445] were implemented for accelerating the CP simulations. Recently, machine learning (ML) methods have gained attention in CP applications due to its ability to accelerate the CP simulations via a function approximation tool, instead of these being explicitly programmed [446]. ML techniques usually depend on trained models which are not computationally demanding, compared to conventional numerical simulations. For instance, Ali et al. [447] proposed a new approach by coupling ANN and CPFEM to predict the flow behavior of Al sheet metal subjected to complex strain paths. Yuan et al. [448] introduced a novel ML-based reduced order CP model to predict the flow behavior of FCC sheet metals. Miyazawa et al. [449] developed a new framework by coupling ML and CP modelling and predicted the mechanical behavior of steel under cyclic loading. Pandey and Pokharel [450] used ML approaches coupled with CP modelling to predict the texture evolution of copper sheets subjected to uniaxial tension. Acar [451] proposed a novel ML-based computational framework to predict CP parameters and revealed the relations of these parameters with the texture of Ti alloy, as depicted in Figure 36. Ibragimova et al. [452] proposed a novel approach to predict the localized deformation in sheet metals by coupling convolutional neural network (CNN) and CPFEM, as depicted in Figure 37, to predict localized deformation in metals.

## 6. Conclusions and Outlook

### 6.1. Conclusions and Remarks

This comprehensive review discussed the characterization and modelling approaches for SMF of lightweight alloys based on the PRISMA guidelines. The following conclusions can be drawn:

- In the past few decades, the numerical methods in SMF simulation have undergone rapid development, including from two-dimensional to three-dimensional, from symmetric to asymmetric, from classical to advanced constitutive models, traditional mesh to meshless, etc.
- Plasticity models under NAFR were widely developed and applied in SMF simulation due to high accuracy, high flexibility, user-friendly parameter identification and convenient consideration of the continuous anisotropic/distortional hardening (yield surface evolution) of lightweight alloys under proportional and non-proportional loadings.
- Advanced CP models can capture the underlying process-microstructure-properties for the physical mechanisms. Besides, applying CP models in predicting the constitutive behaviors of lightweight alloys under complex loading conditions (challenging to achieve in mechanical characterization) has drawn significant attention.
- The application of the DIC technique improved the accuracy of mechanical characterization and promoted the development of advanced characterization techniques, e.g., inverse engineering. Novel mechanical characterization methods under SPCs, rather than reverse loadings, are required for the engineering application of advanced lightweight alloys.
- The formability (FLD) of sheet metals is usually assessed experimentally by Nakajima test (out-of-plane) or Marciniak test (in-plane). Several methods were developed to determine the onset of localized necking, e.g., spatial methods, temporal methods and spatio-temporal methods.
- Theoretical models based upon bifurcation theory, geometrical imperfection theory and continuum damage mechanics were developed to predict the forming limit strains of lightweight alloys under various loading conditions, e.g., strain path changes, through coupling different hardening laws and yield criteria.
- Multiscale forming limit diagram (FLD) prediction schemes were developed by combining the CPFEM with the geometrical imperfection theory, e.g., M–K model. Besides, approaches or frameworks by coupling machine learning and CP simulations were proposed to predict the FLD of lightweight alloys.

### 6.2. Outlook and Future Directions

Despite the extensive knowledge accumulated in characterization and modelling approaches for SMF of lightweight metallic materials, there are still several directions of progressive research that should be continued:

➣ The development and application of advanced multiscale modelling approaches for the simulations of SMF under particular conditions (or extreme manufacturing), such as hydrogen environments, cryogenic temperatures and heterostructures/laminated materials, is notable.
➣ For phenomenological constitutive modelling, these efforts include the development of the NAFR framework via analytical parameter identification. Attention should be further focused on stress state-dependent anisotropy and hardening due to the specific underlying mechanisms, such as phase transformation and slip/twinning activity in advanced lightweight materials.
➣ The inverse engineering method, as the next-generation of mechanical characterization, for identification of the parameters of advanced constitutive models under large plastic strains should be highlighted. The focus should be on a reasonable choice of the optimization algorithm and the constitutive model. A suitable plasticity model with high flexibility and fidelity should be selected appropriately in inverse engineering.
➣ Machine learning-based methods, such as artificial neural network, are drawing more attention. As fundamental inputs to train and test these data-driven models, massive, accurate and representative data are crucial to the parameterized artificial intelligence framework. This requirement dramatically fosters the development of advanced characterization techniques, including mechanical experiments and virtual data generation based on accurate multi-physics models (e.g., CP) for advanced lightweight materials.

## Figures and Tables

**Table 1:** Summary of representative lightweight materials for automobiles. Reused from Ref. [9], open access.

Lightweight Materials	Typical Components	Examples	
		Model	Application
Al alloys	Shock absorber, brake, piston, tank, wheel rim, fender, roof, door, bumper, heat insulator, handle, piping, steering component, conrod, rotor, suspension component, bonnet, chassis, spoke, valve, gas cylinder, seat frame.	Audi A8	Chassis
		Jaguar XE	Monocoque
		Mercedes AMG GT	Body
		Ford F-150	Body panel
		Toyota GT86	Bonnet
		Mazda MX-5	Bumper
		Nissan Leaf	Battery case, sealing component
		Tesla Model S	Frame and heat exchangers
Mg alloys	Engine block, steering wheel frame, seat frame, instrument panel, wheel rim, cylinder head, clutch case, cylinder block, transmission case, lower crankcase, intake manifold, air intake system, steering link bracing, oil pump body, camshaft drive chain case, gear control housing, bracket.	Ford Thunderbird	Steering wheel frame
		Chrysler Plymouth	
		BMW(MINI)	
		Lexus LS430	
		Mercedes Roadster 300/400/500	SL Seat frame
		Lexus LS430	
		Chrysler Jeep	Instrument panel
		Audi A8	
		Toyota Century	
		Toyota 2000GT	Wheel rim
		Toyota Supra	
		Alfa Romeo GTV	
		Porsche AG 911	
		Dodge Raw	Cylinder head
		Volvo Motors (LCP)	
		Honda Motor	
		Volkswagen Passat	Transmission case
		Audi A4, A6	
		Porsche AG 911	
Ti alloys	Connecting rod, engine valve, spring, intake valve, wheel, turbocharger, exhaust system, muffler, body frame, engine rocker arm, suspension component, engine piston pin, fastener, lug nut, door penetration beam, car stop bracket, brake caliper piston, pin bolt, pressure plate, shift button, clutch circle, fuel tank, fuel cell separator.	Mercedes-Benz S-class	Brake guide pin
		Volkswagen	Sealing washer (brake)
		Honda S2000 Roadster	Gear shift knob
		Porsche GT3	Connecting rod
		Toyota Altezza 6cyL	Valve
		Nissan Infinity Q45	
		Mercedes-Benz truck	Turbocharger rotor
		Mitsubishi Lancer	
		GM Corvette Z06	Exhaust system
		Acura NSX	Engine
		Volkswagen Lupo FSI	Suspension spring

**Table 2:** The advanced NAFR constitutive models.

Reference	Non-Quadratic	SD Effect	Analytical Calibration
Stoughton (& Yoon) [146,148]	×	×	√
Stoughton & Yoon [147]	×	√	√
Min et al. [149]	√	×	√
Lee et al. [150]	√	×	√
Chen et al. [154]	√	×	√
Park et al. [151]	√	√	√
Hou et al. [155]	√	√	√
Hu et al. [156]	√	√	√
Lou et al. [153]	√	√	√

**Table 3:** Constitutive models to capture the Bauschinger effect under SPCs.

Modelling Strategy	Back Stress	Change of Yield Surface Shape	References
Kinematic hardening	√	×	[167,168,169,170,172,173,174,175,193]
Distortional hardening	×	√	[166,176,177,178,179,180,181,183,184,186,187,194]
Combined hardening	√	√	[188,189,190,191,192,195,196,197,198]

## Data Availability

Not applicable.
